# Redox-Mediated Inactivation of the Transcriptional Repressor RcrR is Responsible for Uropathogenic Escherichia coli’s Increased Resistance to Reactive Chlorine Species

**DOI:** 10.1128/mbio.01926-22

**Published:** 2022-09-08

**Authors:** Sadia Sultana, Mary E. Crompton, Kennadi Meurer, Olivia Jankiewicz, Grace H. Morales, Colton Johnson, Elise Horbach, Kevin Pierre Hoffmann, Pooja Kr, Ritika Shah, Greg M. Anderson, Nathan T. Mortimer, Jonathan E. Schmitz, Maria Hadjifrangiskou, Alessandro Foti, Jan-Ulrik Dahl

**Affiliations:** a School of Biological Sciences, Microbiology, Illinois State University, Normal, Illinois, USA; b Department of Pathology, Microbiology and Immunology, Division of Molecular Pathogenesis, Vanderbilt University Medical Center, Nashville, Tennessee, USA; c School of Biological Sciences, Cellular Immunology, Illinois State University, Normal, Illinois, USA; d Vanderbilt Institute of Infection, Immunology and Inflammation, Nashville, Tennessee, USA; e Max Planck Institute for Infection Biology, Cellular Microbiology, Berlin, Germany; University of Michigan-Ann Arbor

**Keywords:** bacterial defense systems, hypochlorous acid, oxidative stress, reactive chlorine species, transcriptional regulation

## Abstract

The ability to overcome stressful environments is critical for pathogen survival in the host. One challenge for bacteria is the exposure to reactive chlorine species (RCS), which are generated by innate immune cells as a critical part of the oxidative burst. Hypochlorous acid (HOCl) is the most potent antimicrobial RCS and is associated with extensive macromolecular damage in the phagocytized pathogen. However, bacteria have evolved defense strategies to alleviate the effects of HOCl-mediated damage. Among these are RCS-sensing transcriptional regulators that control the expression of HOCl-protective genes under non-stress and HOCl stress. Uropathogenic Escherichia coli (UPEC), the major causative agent of urinary tract infections (UTIs), is particularly exposed to infiltrating neutrophils during pathogenesis; however, their responses to and defenses from HOCl are still completely unexplored. Here, we present evidence that UPEC strains tolerate higher levels of HOCl and are better protected from neutrophil-mediated killing compared with other E. coli. Transcriptomic analysis of HOCl-stressed UPEC revealed the upregulation of an operon consisting of three genes, one of which encodes the transcriptional regulator RcrR. We identified RcrR as a HOCl-responsive transcriptional repressor, which, under non-stress conditions, is bound to the operator and represses the expression of its target genes. During HOCl exposure, however, the repressor forms reversible intermolecular disulfide bonds and dissociates from the DNA resulting in the derepression of the operon. Deletion of one of the target genes renders UPEC significantly more susceptible to HOCl and phagocytosis indicating that the HOCl-mediated induction of the regulon plays a major role for UPEC’s HOCl resistance.

## INTRODUCTION

Escherichia coli is one of the best and most thoroughly studied free-living organisms. Members of this species are characterized by remarkable diversity: while some E. coli strains live as harmless commensals in mammalian intestines, other distinct genotypes represent serious intestinal pathogens that cause significant morbidity and mortality and are categorized into the six distinct pathotypes (1). Yet another group of life-threatening pathogens are extraintestinal E. coli, including uropathogenic E. coli (UPEC), the most common etiologic agent in approximately 80% of urinary tract infections (UTIs) (2–4). One major difference to intestinal pathogens is that UPEC grow as seemingly harmless commensals in the intestinal environment but rapidly turn into serious pathogens after entry into the urinary tract (5). UPEC ascend from the urethra to the bladder, where they adhere to uroepithelial cells, are internalized, and form biofilm-like bacterial communities in the protected intracellular environment of the host cell (2, 6).

However, prior to their attachment to uroepithelial cells, UPEC must surpass host defense mechanisms, including phagocytic attack by neutrophils (2). Within the phagosome of neutrophils, bacteria are confronted with a complex mixture of antimicrobial compounds, including reactive oxygen and chlorine species (ROS; RCS) (7, 8). Production of neutrophilic ROS and RCS, a process named oxidative burst, involves the assembly and activation of NADPH oxidase. This enzyme complex catalyzes the reduction of molecular oxygen to superoxide in the phagosomal space, which is subsequently dismutated to hydrogen peroxide (H_2_O_2_). Intracellular granules also release myeloperoxidase into the phagosome, an antimicrobial enzyme that converts H_2_O_2_ and available (pseudo-) halides into microbicidal hypohalous acids (9–11). In contrast to H_2_O_2_, which shows only very modest reactivity with most cellular macromolecules and is well tolerated by most bacterial species even at millimolar concentrations (12), hypochlorous acid (HOCl), the most prominent hypohalous acid, is extremely reactive and already bactericidal at low micromolar levels (13, 14). HOCl oxidizes virtually any cellular molecule, including select amino acids, lipids, metal centers, and nucleic acids (15). This, in turn, leads to macromolecular damage and, ultimately, microbial death. One well-known target of HOCl is the amino acid cysteine (8, 9). HOCl or related chloramines oxidize cysteines to either reversible (i.e., sulfenic acids; disulfide bonds) or irreversible oxidative thiol modifications (i.e., sulfinic and sulfonic acid) (16). Reversible thiol modifications often have structural and functional consequences while irreversible thiol modifications can lead to protein aggregation and degradation (12, 13).

Bacteria have evolved various strategies to counteract and reduce the harmful effects of ROS/RCS stress. ROS/RCS significantly affect global gene expression, which often is a result of changes in the activities of redox-regulated transcriptional regulators. Posttranslational modifications of redox-sensitive amino acid side chains in these regulatory proteins affect their promoter DNA binding activity and ultimately the expression of the corresponding stress-protective target genes. Although HOCl is one of the most potent industrial and physiological antimicrobials (17), little is known about the cellular consequences of HOCl stress in Gram-negative pathogens. This is particularly surprising given that bacterial responses to the two less reactive bactericidal oxidants H_2_O_2_ and superoxide have been studied in great detail (18–22). Many of their responses involve select transcriptional regulators, which can distinguish between different stressors through oxidation of conserved cysteine residues (23). So far, three HOCl-sensing transcriptional regulators have been identified and all of them in the K-12 strain MG1655: HypT, which is activated through methionine oxidation (24); and the TetR-family repressor NemR and the AraC-family activator RclR, which represent two transcription factors that use the oxidation status of cysteine residues to sense and respond to HOCl. Oxidation of NemR leads to its dissociation from the promoter, causing derepression of its target genes (25). In contrast, the transcriptional activator RclR binds its target DNA upon HOCl-mediated cysteine oxidation, leading to a strong and specific activation of the expression of the *rclABC* operon (26).

It is well accepted that neutrophils and the oxidative burst play a crucial role for the clearance of UPEC during UTI (27). Previous studies have identified defense systems against hydrogen peroxide (H_2_O_2_) that positively affect UPEC’s ability to colonize the bladder emphasizing the importance of bacterial oxidative stress defense systems for UPEC pathogenesis (28–30). However, these defense systems are also present in commensal E. coli, which limits their suitability as UPEC-specific drug targets. Here, we demonstrate for the first time that resistance to the most abundant neutrophilic oxidant, HOCl, and the oxidizing environment of the neutrophil phagosome is significantly higher in UPEC compared with other E. coli pathotypes, indicating the presence of at least one additional HOCl defense system. Intriguingly, our knowledge on UPEC’s HOCl defense systems is quite limited. Our transcriptomic data show that UPEC responds to sublethal HOCl-stress with the upregulation of an operon harboring three uncharacterized genes that are not present in the nonpathogenic E. coli strain MG1655. We identified one of them as a HOCl-responsive transcriptional repressor, RcrR, that reversibly loses its repressor activity during HOCl-stress. The thiol-based inactivation mechanism of RcrR is based on intermolecular disulfide bond formation, which likely results in conformational changes and disables the repressor binding to the promoter DNA. RcrR’s inactivation results in the derepression of the downstream targets, one of which we identified as a major contributor to UPEC’s increased resistance to HOCl-stress and neutrophil-mediated killing.

## RESULTS

### UPEC shows increased growth and survival during HOCl stress.

**FIG 1:**
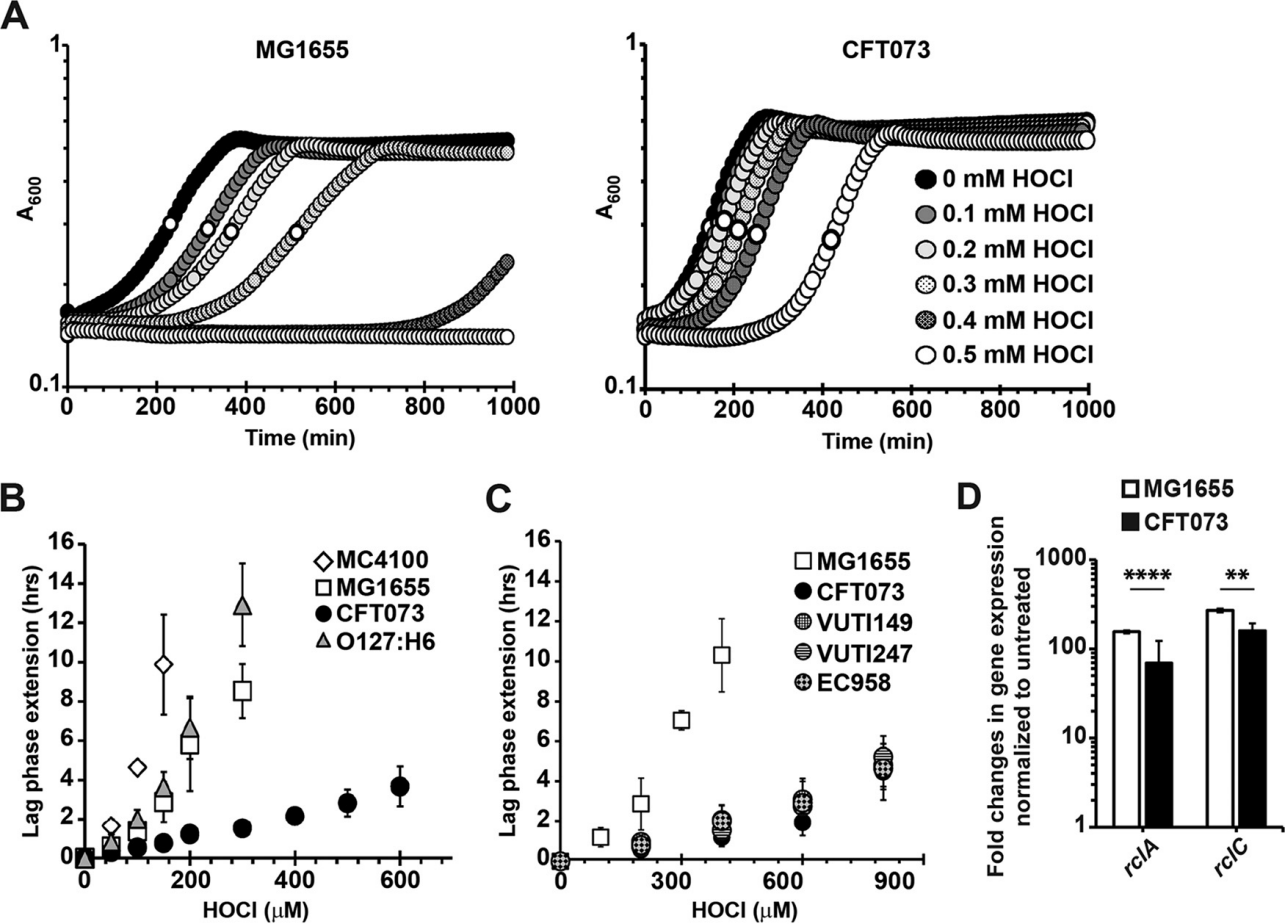
UPEC strains show increased protection from HOCl stress compared with strains from other E. coli pathotypes. (A to C) Different E. coli strains were cultivated aerobically in MOPSg media in the presence of the indicated concentrations of HOCl. Growth was monitored for 16 h at 600 nm. (A) Treatment with sublethal HOCl concentrations causes a concentration-dependent lag phase extension (LPE) in strains MG1655 and CFT073. This effect is less pronounced in CFT073 indicating its increased HOCl resistance. (B and C) HOCl-mediated LPE was calculated for each strain (see Materials and Methods for a detailed protocol). (B) LPE of HOCl-stressed E. coli strains MG1655 (white squares) and MC4100 (white diamonds) as well as enteropathogenic E. coli O127:H6 (gray triangle) were significantly increased compared with UPEC strain CFT073 (black circles). (*n* = 7, ± SD). (C) All four UPEC strains (circles) showed higher HOCl resistance than the nonpathogenic strain MG1655 (white square) (*n* = 3, ± SD). (D) Induction of *rclA* and *rclC* expression was determined by qRT-PCR in HOCl-treated CFT073 (black bar) and MG1655 (white bar). The expression of both genes was significantly reduced in CFT073 compared with MG1655. Student’s *t* test (GraphPad Prism): ** 0.01 > *P* > 0.001; **** *P* < 0.0001; (*n* = 3, ± SD).

In the bladder, UPEC is confronted with an onslaught of host defense mechanisms, including exposure to neutrophilic oxidants (31). Given that UPEC appears to thrive in an environmental with increased levels of antimicrobial oxidants, we speculated that extraintestinal E. coli may tolerate higher HOCl levels than intestinal E. coli pathotypes. To test this possibility, we compared the growth behavior of different E. coli strains during HOCl stress. To reduce the possibility that media components react with and potentially quench HOCl, we performed our phenotypic assays in MOPS-glucose (MOPSg) minimal media. We cultivated the two genetically distinct E. coli strains MG1655 and CFT073 and monitored their growth for 16 h in the presence and absence of increasing HOCl concentrations. MG1655 is a fecal isolate and was adapted to one of the most robust E. coli lab strains (32). CFT073, on the other hand, is a uropathogen that was isolated from the blood of a patient with acute pyelonephritis (33). Exposure of MG1655 and CFT073 to increasing HOCl concentrations resulted in concentration-dependent extensions of their lag phase (LPE) (Fig. 1A). We found HOCl-mediated LPE are more pronounced in MG1655 indicating a higher HOCl susceptibility of that strain compared with CFT073. We then used the growth curve-based assay to compare the sensitivity of additional E. coli strains to sublethal HOCl stress by quantifying their HOCl-mediated changes in LPE. This method has previously been found to be most reproducible for assessing HOCl sensitivity (34). We confirmed the drastically higher HOCl sensitivity of MG1655 while the HOCl concentrations tested had only minor effects on the LPE of CFT073 (Fig. 1B). This is significant given that MG1655 is considered much more HOCl-resistant than most other nonpathogenic laboratory E. coli strains, including MC4100, which lacks a 97 kb region encompassing the complete RclR regulon (35). The HOCl-sensing transcriptional regulator RclR activates the expression of *rclA*, *rclB*, and *rclC*, all of which contribute to HOCl resistance (26). Our LPE data confirmed that MC4100 is indeed less HOCl-tolerant than MG1655 (Fig. 1B). No significant difference in HOCl sensitivity was observed between MG1655 and the enteropathogenic E. coli strain O127:H6, the first E. coli pathovar causing infantile diarrhea (36) (Fig. 1B). To exclude the possibility that the higher HOCl resistance of CFT073 is a strain-specific phenomenon, we analyzed the growth behavior of additional UPEC strains during HOCl stress, including the dominant fluoroquinolone-resistant clone EC958 (37) and two clinical isolates from cystitis patients, VUTI149 and VUTI247 (38). Notably, all four UPEC strains showed similar LPE responses in the presence of HOCl and were substantially more resistant than K-12 strain MG1655 (Fig. 1C). In contrast, MG1655 and CFT073 displayed similar sensitivity to H_2_O_2_, another neutrophilic oxidant generated during phagocytosis, excluding the possibility that UPEC’s increased resistance is targeted to ROS/RCS in general (Fig. S1). We conclude from these data that the increased HOCl resistance is potentially characteristic for the UPEC pathotype in general.

10.1128/mbio.01926-22.1 FIG S1 Effects of hydrogen peroxide (H_2_O_2_) on the growth of E. coli strains MG1655 and CFT073. Growth phenotype analyses of CFT073 and MG1655 were performed in MOPSg media in the presence of the indicated concentrations of H_2_O_2_. H_2_O_2_-mediated LPE was calculated for each strain (see Materials and Methods for a detailed protocol). No significant differences in H_2_O_2_-induced LPE were observed between the two strains (*n* = 4, ± SD). Download FIG S1, TIF file, 2.6 MB. Copyright © 2022 Sultana et al. 2022 Sultana et al. https://creativecommons.org/licenses/by/4.0/ This content is distributed under the terms of the Creative Commons Attribution 4.0 International license.

### HOCl causes extensive transcriptional changes in UPEC.

In contrast to E. coli MG1655, the HOCl stress response of UPEC strains is still completely unexplored. As a first test to determine whether HOCl-stressed CFT073 and MG1655 share similarities in gene expression, we determined the sublethal HOCl concentrations for each strain and then used quantitative real-time PCR (qRT-PCR) to compare the HOCl-induced expression of the *rclC* and *rclA* genes. Both genes are members of the RclR regulon, the expression of which is induced in HOCl-stressed MG1655 (26). We confirmed the transcriptional upregulation of both genes in MG1655 and found that their expression was also significantly induced in CFT073 although the changes in *rclA/rclC* expression level were significantly lower compared with MG1655 (Fig. 1D). These data suggest UPEC may employ at least one additional HOCl defense system, which is not present in EPEC or nonpathogenic E. coli and which, at least in part compensates for UPEC’s upregulation of the RclR regulon under HOCl stress.

**FIG 2:**
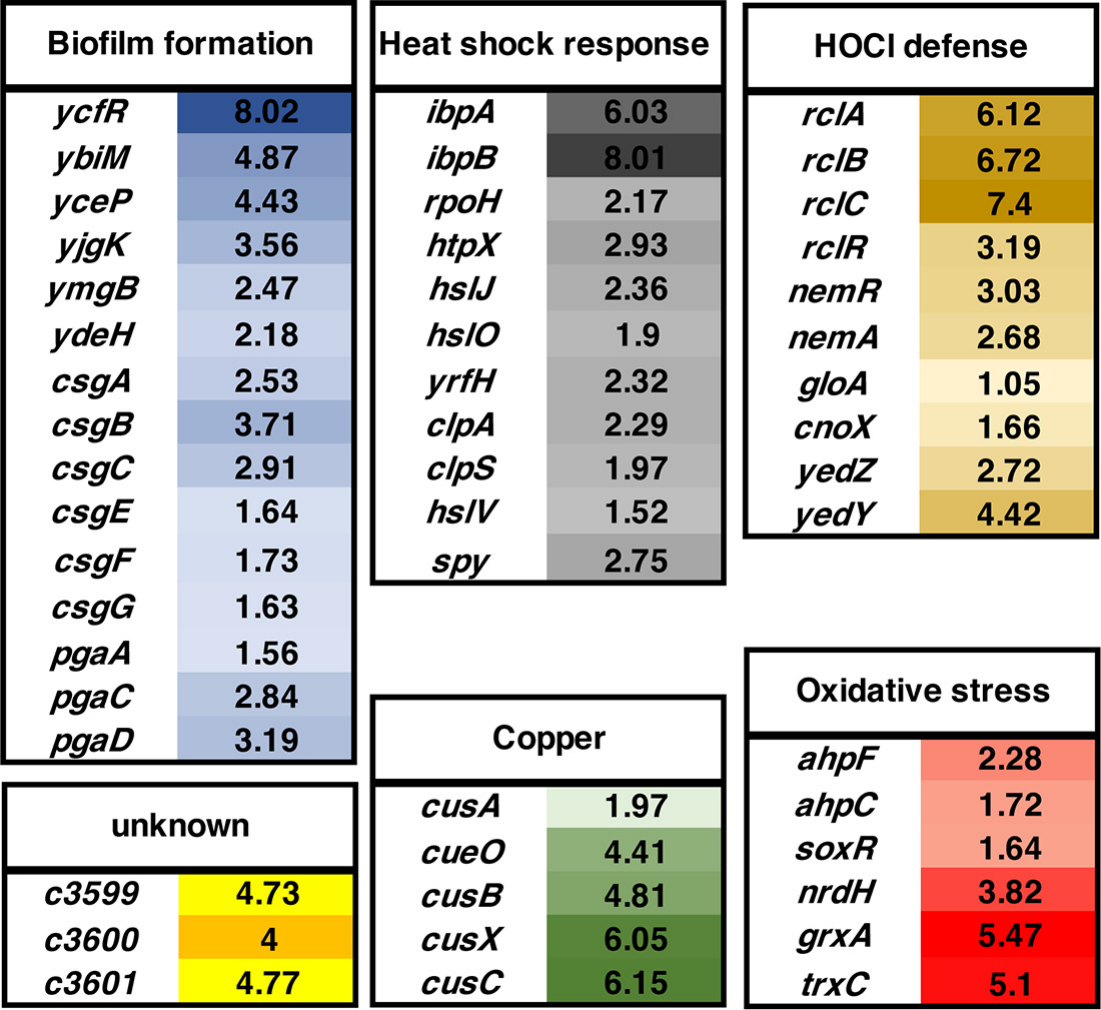
Global gene expression changes in UPEC strain CFT073 in response to sublethal HOCl stress. Exponentially growing CFT073 cells were incubated with a sublethal concentration of HOCl (2.25 mM) for 15 min. Transcription was stopped by the addition of ice-cold methanol. Reads were aligned to the CFT073 reference genome (Accession number: AE014075). Log_2_-fold changes of the expression of select CFT073 genes in HOCl-stressed CFT073 cells relative to untreated CFT073 sorted by biological function. The color intensities correlate with the degree of upregulation in gene expression.

Next, we conducted RNAseq analysis to globally monitor changes in CFT073 gene expression in response to sublethal HOCl stress. CFT073’s chromosome shows a mosaic structure in the distribution of backbone genes, which are conserved between MG1655 and CFT073, and “foreign” genes that presumably have been acquired horizontally (5). Genomic comparisons of different UPEC strains, including CFT073, revealed that they share more than 80% of their open reading frames but only approximately 40% of their genome is also found in the K-12 strain MG1655 (33). We, therefore, reasoned that any CFT073 genes that are highly upregulated upon HOCl stress and that are either not present or not significantly overexpressed in HOCl-treated MG1655 (25) could contribute to CFT073’s elevated HOCl resistance. For the transcriptome analysis, we included all genes annotated in the NCBI database and compared the expression values of the stress treated cells to non-stress treated controls. We set a false discovery rate (FDR) of <0.005 as a threshold for significance and considered transcripts as upregulated when they showed a log_2_-fold change of >1.5, and downregulated when they showed a log_2_-fold change of <-1.5. Treatment of CFT073 with 2.25 mM HOCl for 15 min caused the upregulation of 757 genes and downregulation of 681 genes, most of which have an unknown function (Table S1). While 32% of these genes are also differentially expressed in HOCl-stressed MG1655 (25), the remaining 68% are either UPEC-specific genes or not induced by HOCl in MG1655 making them of particular interest for us. Likely due to the proteotoxic nature of HOCl (39), members of the heat shock response were among the most upregulated genes in HOCl-stressed CFT073 cells, including genes that encode proteases and the molecular chaperones IbpA, IbpB, Spy, and HslO/Hsp33, respectively (Fig. 2). Not surprisingly and consistent with previous studies we also identified antioxidant (e.g., *grxA*, *trxC*, *ahpCF*) and copper resistance systems (e.g., *cusABCX*, *cueO*) in the group of highly upregulated genes (18, 25, 34). Moreover, the expression of four previously characterized HOCl-stress defense systems was increased in CFT073 upon HOCl exposure (i.e., *rclRABC*, *nemRA*, *cnoX*, *yedYZ*) (25, 26, 40–42). Our RNAseq analysis also revealed the upregulation of many biofilm genes in HOCl-stressed CFT073 (Fig. 2; Table S1). These include the curli-producing *csgABC* and *csgEFG* operons, the diguanylate cyclase encoding gene *ydeH*, the biofilm stress resistance gene *ycfR*, and the *pgaABCD* operon, which produces the major adhesin poly-β-1,6-N-acetylglucosamine (poly-GlcNAc) and represents a significant contributor to UPEC’s virulence *in vivo* (43, 44).

10.1128/mbio.01926-22.7 TABLE S1 Differentially expressed genes in HOCl-treated CFT073 compared with untreated. Download Table S1, XLSX file, 0.5 MB. Copyright © 2022 Sultana et al. 2022 Sultana et al. https://creativecommons.org/licenses/by/4.0/ This content is distributed under the terms of the Creative Commons Attribution 4.0 International license.

### The CFT073 RcrR regulon confers resistance to HOCl stress.

**FIG 3:**
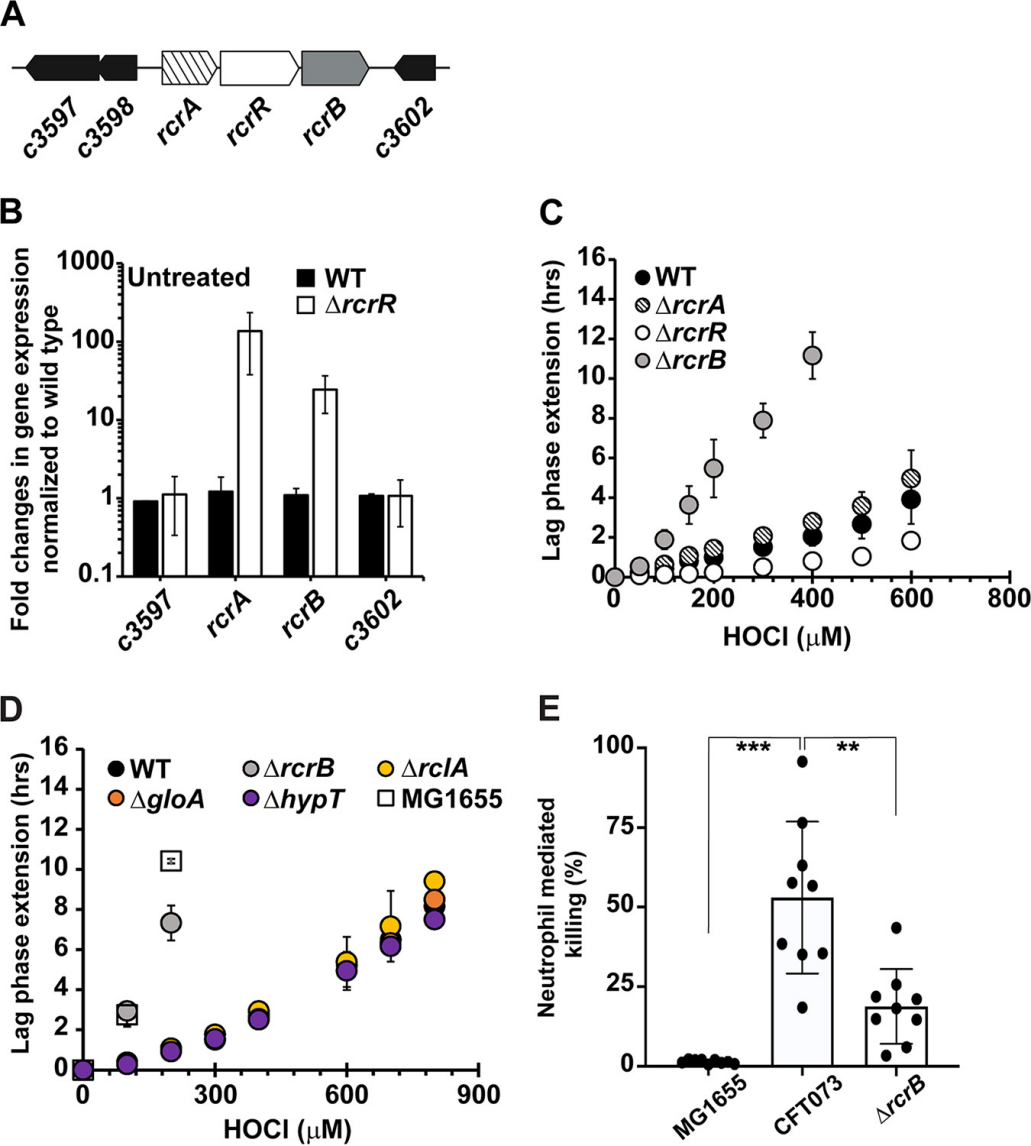
The gene cluster consisting of *rcrA-rcrR-rcrB* protects UPEC strain CFT073 from HOCl stress. (A) Illustration of the gene region containing the *c3599/rcrA*, *c3600/rcrR*, and *c3601/rcrB* loci. (B) CFT073 wild-type (black bars) and Δ*rcrR* cells (white bars) were grown to mid-log phase under nonstress conditions and changes in the expression of the indicated genes were determined by qRT-PCR. *rcrA* and *rcrB* mRNA level were elevated in the Δ*rcrR* strain while expression of *c3597* and *c3602* remained unaffected (*n* = 3, ± SD). (C) Growth phenotype analyses of UPEC strains CFT073 wild-type, Δ*rcrA*, Δ*rcrR*, Δ*rcrB*, and the nonpathogenic E. coli strain MG1655 were performed in MOPSg media in the presence of the indicated HOCl concentrations. HOCl-mediated LPE was calculated for each strain (see Materials and Methods for a detailed protocol). Deletion of the *rcrR* gene rendered CFT073 more resistant whereas *rcrB*-deficient cells were substantially more sensitive to HOCl compared with the wild-type (*n* = 5, ± SD). (D) Growth phenotype analyses of UPEC strains CFT073 wild-type, Δ*rcrB*, Δ*rclA*, Δ*gloA*, Δ*hypT*, and the nonpathogenic E. coli strain MG1655 were performed in MOPSg media in the presence of the indicated HOCl concentrations. HOCl-mediated LPE was calculated for each strain (see Materials and Methods for a detailed protocol). (*n* = 3, ± SD). (E) Serum-opsonized E. coli strains MG1655, CFT073, and Δ*rcrB* were incubated with neutrophils isolated from human blood at MOI of 10:1 for 60 min at 37°C. Killing of each strain was determined by plating on LB agar for CFU. UPEC strain CFT073 was 54% more resistant to neutrophil-mediated killing than the nonpathogenic E. coli strain MG1655, while Δ*rcrB* showed only 17% resistance compared to MG1655. Two-way ANOVA (GraphPad Prism): *** *P* = 0.0002; ** *P* = 0.0011 (*n* = 3 [with three technical replicates each], ± SD).

Including a gene cluster consisting of the three genes, *c3599*, *c3600*, and *c3601*, 32.4% of the differentially expressed genes identified in our RNAseq analysis are uncharacterized and their biological function is still unknown (Fig. 2; Table S1). The *c3599-c3600-c3601* gene cluster is divergently transcribed from the genes located directly upstream and downstream (i.e., *c3597* and *c3602*, respectively) (Fig. 3A). *c3600* is located downstream of *c3599* and upstream of *c3601* and encodes a putative TetR-family transcriptional regulator; however, the functions of the *c3599* and *c3601* genes are unknown (Fig. 3A). We renamed the *c3599*, *c3600*, and *c3601* genes to *rcrA*, *rcrR*, and *rcrB*, respectively, to reflect their role in **r**eactive **c**hlorine species **r**esistance. qRT-PCR analysis of HOCl-stressed CFT073 cells confirmed that the transcript levels of these genes are indeed elevated (Fig. S2A). To determine whether RcrR also functions as a transcriptional repressor and to identify potential genes under its control, we performed gene expression studies in CFT073 wild-type and *ΔrcrR* cells that were grown to mid-log phase under non-stress conditions. qRT-PCR analysis revealed that in comparison with CFT073 wild-type, expression of *rcrA* was 105-fold increased while *rcrB* mRNA level were 24-fold higher in the absence of the *rcrR* gene (i.e., Δ*rcrR*) (Fig. 3B). These data indicate RcrR represses both genes under nonstress conditions and that the HOCl-mediated upregulation of *rcrA/rcrB* likely depends on RcrR’s dissociation from the promoter resulting in the transcription of both genes. In contrast, expression of *c3597* and *c3602* was not affected by the presence or absence of *rcrR* (Fig. 3B). RNA-seq analysis of Δ*rcrR* cells under nonstress conditions confirmed the constitutive expression of *rcrA* and *rcrB* and indicated that the presence of RcrR also affects the expression of additional genes most of which are part of the CysB regulon (Table S2).

10.1128/mbio.01926-22.2 FIG S2 The upregulation of the RcrR regulon plays a role for UPEC’s survival during exposure to HOCl but not to H_2_O_2_. (A) Upregulation of the expression of *rcrA*, *rcrR*, and *rcrB* upon treatment of CFT073 with 2.5 mM HOCl was determined by qRT-PCR. The expression of all three genes was significantly induced in HOCl-stressed cells (*n* = 4, ± SD). (B) Strains CFT073, *ΔrcrR*, and *ΔrcrB* were grown in MOPSg media until the OD_600 nm_ = 0.5 to 0.55 was reached. Cells were then either left untreated (–) or treated with 3 mM HOCl (+), incubated for 30 min, 10-fold serial diluted, spotted onto LB agar plates, and incubated over night at 37°C. In comparison with wild-type CFT073, survival of *ΔrcrR* was ~1 log more resistant, whereas no survival was detected for *rcrB*-deficient cells. The phenotype was verified in three independent experiments. (C) CFT073 and *ΔrcrR* cells were cultivated aerobically at 37°C in MOPSg media in the presence of the indicated concentrations of H_2_O_2_. LPE was calculated for each strain (see Materials and Methods for a detailed protocol). No significant differences in LPE were observed between the two strains at the concentrations tested (*n* = 3, ± SD). Download FIG S2, TIF file, 1.8 MB. Copyright © 2022 Sultana et al. 2022 Sultana et al. https://creativecommons.org/licenses/by/4.0/ This content is distributed under the terms of the Creative Commons Attribution 4.0 International license.

10.1128/mbio.01926-22.8 TABLE S2 Differentially expressed genes in Δ*rcrR* cells compared with CFT073 wild-type. Download Table S2, XLSX file, 0.2 MB. Copyright © 2022 Sultana et al. 2022 Sultana et al. https://creativecommons.org/licenses/by/4.0/ This content is distributed under the terms of the Creative Commons Attribution 4.0 International license.

To determine whether any of the three genes play a role for CFT073’s HOCl resistance, we constructed strains with individual in-frame gene deletions and compared their HOCl sensitivities to wild-type cells using the LPE assay. Deletion of *rcrA* (i.e., Δ*rcrA*) did not significantly affect the LPE at the HOCl concentrations tested (Fig. 3C). However, we found that CFT073 cells lacking the *rcrB* gene (i.e., Δ*rcrB*) were highly susceptible to HOCl (Fig. 3C). Remarkably, the Δ*rcrB* strain was equally sensitive to HOCl as the commensal E. coli strain MG1655, suggesting that expression of *rcrB* is the main contributor to UPEC’s increased HOCl tolerance *in vitro*. In contrast, deletion of the *rcrR* gene (i.e., Δ*rcrR*) caused a significant decrease in HOCl-induced LPE indicating that constitutive overexpression of the operon provides additional protection against HOCl (Fig. 3C). Consistently, we found that survival of Δ*rcrR* was ~1 log higher than wild-type CFT073 after 30 min exposure to 3 mM HOCl, while no survival was detected for the Δ*rcrB* strain (Fig. S2B). No difference in LPE was observed when CFT073 wild-type and Δ*rcrR* were exposed to H_2_O_2_ (Fig. S2C). To compare RcrB-mediated protection of CFT073 from HOCl stress with HOCl defense systems that were previously identified in MG1655 but are also present in UPEC strain CFT073, we created gene deletion mutants in the CFT073 background that are defective in *hypT* (HOCl-sensing transcriptional regulator), *gloA* (member of NemR regulon), and *rclA* (member of the RclR regulon), respectively. We chose these genes because their deletion resulted in the most prominent loss in survival in HOCl-treated MG1655 compared with other members of the regulon (24–26). We confirmed the growth delays of strains MG1655 and CFT073Δ*rcrB*. In contrast, deletion of *rclA*, *gloA*, or *hypT* had no significant effect on the growth of CFT073 (Fig. 3D) indicating that RcrB is the major HOCl defense system in CFT073.

Next, we investigated whether MG1655, CFT073, and Δ*rcrB* also differ in their sensitivity to phagosomal killing by neutrophils, which generate HOCl as the major reactive species during oxidative burst (7). Freshly isolated neutrophils were incubated for 45 min with a 10-fold excess of opsonized MG1655, CFT073, and Δ*rcrB*, respectively. Neutrophils and phagocytized bacteria were then separated from noningested bacteria and plated for CFU counts after lysis of the neutrophils. We found that CFT073 was ~54% more resistant to phagocytosis than E. coli MG1655 (Fig. 3E), suggesting that a higher HOCl resistance could play a role for UPEC’s ability to survive neutrophil infiltration and establish disease. The absence of *rcrB* (i.e., Δ*rcrB*) reduces CFT073’s ability to counter neutrophil attack to only 17% (Fig. 3E), indicating a critical role of RcrB during phagocytosis.

**FIG 4:**
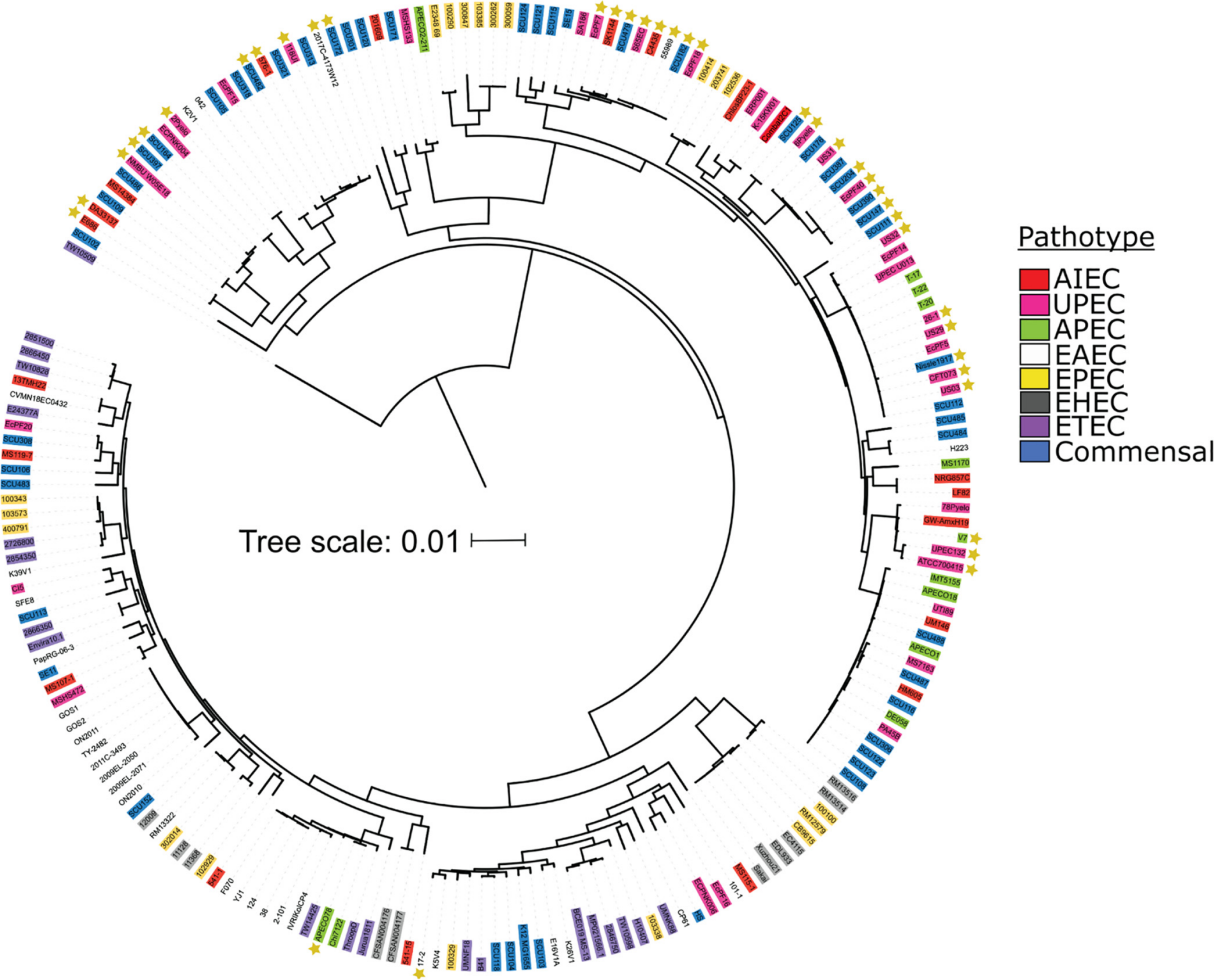
Distribution of *the rcrARB* operon in different E. coli pathotypes. Genomes from 196 E. coli strains of eight pathotypes were downloaded from NCBI and a custom BLAST database was created. A core genome alignment was constructed using Roary version 3.13.0, and a maximum likelihood tree built using IqTree version 2.1.4_beta. The tree was visualized and annotated using Interactive Tree of Life with the pathotype and operon presence. E. coli pathotypes used for comparison: AIEC, adherent-invasive E. coli; APEC, avian pathogenic E. coli; EAEC, enteroaggregative E. coli*;* EHEC, enterohemorrhagic E. coli*;* EPEC, enteropathogenic E. coli; ETEC, enterotoxigenic E. coli; commensal E. coli; UPEC, uropathogenic E. coli. The yellow star indicates the presence of the operon, which was lacking in EHEC, EPEC, and ETEC and present in 25% of AIEC, 8% of APEC, 10% EAEC, 22% of commensals, and 46% of UPEC.

We searched for the presence of the *rcrARB* operon in the genomes of 196 E. coli strains from eight distinct pathotypes. While the *rcrARB* gene cluster has not been found in enterohemorrhagic E. coli (EHEC), enteropathogenic E. coli (EPEC), and enterotoxigenic E. coli (ETEC) strains, it was present in 25% of adherent-invasive E. coli (AIEC), 8% of avian pathogenic E. coli (APEC), 10% enteroaggregative E. coli (EAEC), 22% of commensals, and 46% of UPEC (Fig. 4; Fig. S3; Table S3). The highly HOCl-sensitive intestinal E. coli strains MC4100, MG1655, and O127:H6 (Fig. 1B) lack the presence of the *rcrARB* gene cluster. Taken together, our results indicate we have discovered a novel HOCl stress defense system, which is present in multiple E. coli pathotypes and consists of the HOCl-sensing transcriptional repressor RcrR and its regulatory target RcrB, an important contributor to UPEC’s HOCl resistance.

10.1128/mbio.01926-22.3 FIG S3 Distribution of *rcrA-rcrR-rcrB* operon in 196 E. coli strains of eight pathotypes. AIEC, adherent-invasive E. coli; APEC, avian pathogenic E. coli; EAEC, enteroaggregative E. coli*;* EHEC, enterohemorrhagic E. coli*;* EPEC, enteropathogenic E. coli; ETEC, enterotoxigenic E. coli; UPEC, uropathogenic E. coli. The *rcrARB* operon was not present in EHEC, EPEC, and ETEC and present in 25% of AIEC, 16% of APEC, 10% EAEC, 22% of commensals, and 48% of UPEC. Download FIG S3, TIF file, 2.9 MB. Copyright © 2022 Sultana et al. 2022 Sultana et al. https://creativecommons.org/licenses/by/4.0/ This content is distributed under the terms of the Creative Commons Attribution 4.0 International license.

10.1128/mbio.01926-22.9 TABLE S3 Accession numbers of E. coli strains of different pathotypes used for the phylogenetic analysis. Download Table S3, XLSX file, 0.02 MB. Copyright © 2022 Sultana et al. 2022 Sultana et al. https://creativecommons.org/licenses/by/4.0/ This content is distributed under the terms of the Creative Commons Attribution 4.0 International license.

### DNA binding of RcrR is inhibited by reversible thiol oxidation under HOCl stress *in vitro*.

**FIG 5:**
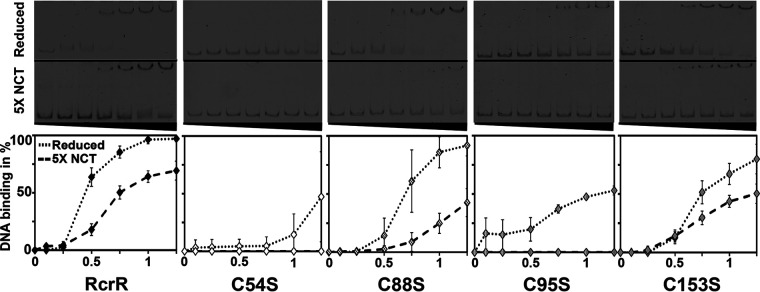
The DNA binding activity of RcrR is inhibited by thiol oxidation after treatment with RCS *in vitro*. Binding of RcrR protein variants (0 to 1.25 μM) to the 440 bp DNA fragment containing the *rcrARB* promoter region (P*_rcrARB_*, 2 ng) in their reduced states (dotted line) and after treatment with a 5-fold excess of N-chlorotaurine (NCT; dashed line) was analyzed in EMSAs. DNA-binding was visualized by 6% TBE-PAGE and assessed by densitometric quantification using ImageJ. DNA binding ability of all the variants was reduced upon oxidation with NCT. DNA binding of RcrR variants C54S and C95S was impaired even in their reduced state and completely abolished after NCT treatment. Representative gels are shown along with the results of the quantification (*n* ≥ 3, ± SD).

To assess the ability of RcrR to bind to the operator sequence upstream of the *rcrA* gene, we purified wild-type RcrR and conducted electrophoretic mobility shift assays (EMSA) using a 440 bp DNA fragment that contains the promoter region of the *rcrARB* operon. We found that reduced wild-type RcrR binds with high affinity to the *rcrARB* upstream region *in vitro* (Fig. 5). Next, we analyzed the DNA-binding ability of RcrR after oxidation with N-chlorotaurine (NCT), a mild, long-lived oxidant generated in innate immune cells as a result of HOCl’s reaction with taurine (13). Here, we used NCT to limit the risk of protein carbonyl formation and protein aggregation (45). NCT treatment substantially decreased the DNA-binding activity of RcrR (Fig. 5), which could be reversed with the thiol-reducing agent dithiothreitol (DTT) (Fig. S4A), suggesting that RcrR senses RCS stress via a reversible oxidative cysteine modification. Not surprisingly and consistent with our *in vivo* data (Fig. S1; Fig. S2C), pretreatment with H_2_O_2_ had no effect on RcrR’s DNA-binding ability (Fig. S4B).

10.1128/mbio.01926-22.4 FIG S4 The DNA binding activity of RcrR is inhibited by reversible thiol oxidation after treatment with HOCl but not with H_2_O_2_. Purified RcrR wild-type (0 to 1.25 μM), in its reduced state and after treatment with a 5-fold excess of (A) N-chlorotaurine (NCT) or (B) H_2_O_2_, was incubated with a 440 bp DNA fragment containing the *rcrARB* promoter (P*_rcrARB_*, 2 ng). To test the reversibility of RcrR’s inactivation, NCT-oxidized RcrR was treated with 2 mM dithiothreitol (DTT) for 15 min prior to incubation with DNA. DNA-binding was assessed by 6% TBE-PAGE and densiometric quantification using ImageJ (*n* ≥ 3, ± SD). Download FIG S4, TIF file, 1.6 MB. Copyright © 2022 Sultana et al. 2022 Sultana et al. https://creativecommons.org/licenses/by/4.0/ This content is distributed under the terms of the Creative Commons Attribution 4.0 International license.

To examine the role of RcrR’s four cysteines in DNA-binding and RCS-sensing, we constructed four His_6_-tagged RcrR variants, in which one of four cysteine residues is individually replaced by serine (i.e., C54S, C88S, C95S, C153S, respectively), and tested their DNA binding activity in the presence and absence of NCT. DNA-binding of RcrR variants C88S and C153S were comparable to the wild-type protein (Fig. 5). In contrast, the binding of the variants C54S and C95S was severely compromised even under reducing conditions and completely abolished when the proteins were pretreated with NCT suggesting that replacement of these cysteines may cause conformational changes that negatively affect DNA-binding. These results support our *in vivo* studies and indicate that RCS-mediated oxidation of RcrR causes its dissociation from the promoter and results in the derepression of *rcrA/rcrB*.

### RcrR responds to HOCl stress by intermolecular disulfide bond formation.

**FIG 6:**
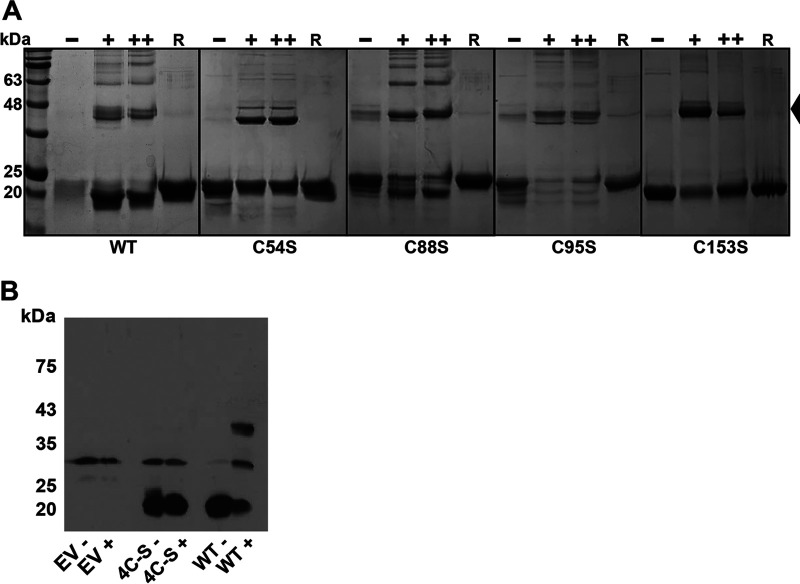
RcrR forms reversible intermolecular disulfide bonds upon exposure to HOCl. (A) 10 μM purified RcrR variants were either left untreated (–) or treated with a 5- (+) and 10- (++) molar ratio of HOCl for 15 min. Proteins were separated by nonreducing SDS-PAGE and visualized by Coomassie staining. The formation of dimers (indicated by the arrow) and higher oligomers was observed in all five HOCl-treated RcrR protein variants. Disulfide bond formation could be reversed by addition of 2 mM DTT (R). Results were verified in four independent experiments. (B) E. coli BL21(DE3) expressing His_6_-tagged RcrR variants wild-type, 4C-S, and the empty expression vector (EV) pET28a were grown to mid-log phase, induced with 100 μM isopropyl 1-thio-β-d-galactopyranoside for 60 min, and then either left untreated (–) or treated with 2.5 mM HOCl (+) for another 15 min. Cells were harvested and reduced cysteines irreversibly alkylated with iodoacetamide. RcrR was visualized by Western blotting using nonreducing SDS-PAGE. Results were verified in three independent experiments.

Given the reversible nature of RcrR’s dissociation from the promoter, we excluded the possibility that irreversible cysteine modifications such as sulfinic (–SO_2_H) and sulfonic acids (–SO_3_H) are formed during RCS treatment. We then wondered whether the RCS-mediated inactivation of RcrR’s repressor activity is a result of intermolecular disulfide bond formation, a reversible cysteine modification associated with redox-signaling. We treated purified RcrR wild-type and the four variant proteins with 5- and 10-molar excess of HOCl prior to separation by nonreducing SDS-PAGE. In the absence of HOCl, all protein variants migrated primarily in their monomeric form (~21 kDa), although some disulfide-bonded species were detected in untreated C88S and C95S variants (Fig. 6A). Upon treatment with HOCl, all variant proteins formed disulfide-linked dimers and intermolecularly disulfide-bonded oligomers, which migrated at ~43, and 65 kDa, respectively, and could be reversed by the addition of DTT. No such disulfide-bonded species were formed after incubation of wild-type RcrR with H_2_O_2_ (Fig. S5A). Based on these data, we hypothesized that a RcrR variant lacking all four cysteine residues (RcrR-4C-S) should not be able to form intersubunit disulfides. Our attempts to purify the RcrR-4C-S variant were not successful, potentially due to their crucial role in protein folding. To confirm the formation of the intermolecular disulfides in RcrR *in vivo*, we performed thiol trapping experiments. We individually expressed the plasmid-encoded His_6_-RcrR variants in mid-log BL21(DE3) cells, exposed them to HOCl, and separated them by nonreducing SDS-PAGE followed by immunodetection of His_6_-RcrR. The ~30 kDa band appears to be unspecific as it was also detected in control cells that only carry the empty vector (Fig. 6B). Disulfide-linked dimer formation (~43 kDa) was observed for HOCl-treated cells that express wild-type protein (Fig. 6B) as well as in variants with individual cysteine substitutions (data not shown). In contrast, the 4C-S variant did not respond to HOCl treatment and remained monomeric (~21 kDa) (Fig. 6B). Coomassie-stained nonreducing SDS gels confirmed the monomeric nature of the 4C-S variant after HOCl-treatment, while the ~21 kDa band disappeared in HOCl-treated cells expressing the wild-type protein (Fig. S5B). Our results indicate the HOCl-responsive mechanism of RcrR is cysteine-dependent even though none of the cysteine residues appears to play the primary role for disulfide-bond formation under the conditions tested. A comparison of RcrR homologs from 96 E. coli strains revealed that all homologs contain the four cysteine residues. However, RcrR homologs also exist in other bacterial pathogens such as Serratia marcescens, Klebsiella pneumoniae, and Salmonella enterica. We extracted the amino acid sequence of RcrR homologs from 31 different bacterial species other than E. coli using NCBI BLAST and visualized the CLUSTAL-W alignment using weblogo plot (Fig. S6). Cys54, Cys88, and Cys95 are conserved among the bacterial species while Cys153 is not.

10.1128/mbio.01926-22.5 FIG S5 (A) 10 μM purified wild-type RcrR was either left untreated (–) or treated with a 5- (+) or 10- (++) molar ratio of H_2_O_2_ for 15 min. Proteins were separated by nonreducing SDS PAGE and visualized by Coomassie staining. No dimer or higher oligomer formation was observed. Results were verified in three independent experiments. (B) Cell lysis samples used for western blot analysis after nonreducing SDS-PAGE and Coomassie staining. Briefly, E. coli BL21(DE3) expressing the His_6_-tagged RcrR variants wild-type, 4C-S, and the empty expression vector (EV) were grown to mid-log phase and induced with 100 μM isopropyl 1-thio-β-D-galactopyranoside for 60 min prior to the treatment with (+) or without (–) 2.5 mM HOCl for another 15 min. Cells were harvested, lysed, and reduced cysteines irreversibly alkylated with iodoacetamide before separating by 12% nonreducing SDS-PAGE. The arrow indicates the presence of the 21 kDa monomer of RcrR. Results were verified in three independent experiments. Download FIG S5, TIF file, 2.7 MB. Copyright © 2022 Sultana et al. 2022 Sultana et al. https://creativecommons.org/licenses/by/4.0/ This content is distributed under the terms of the Creative Commons Attribution 4.0 International license.

10.1128/mbio.01926-22.6 FIG S6 The Weblogo plot of RcrR homologs was constructed to visualize the conserved cysteine residues. Amino acid sequences of RcrR homologs from 31 different bacterial strains were extracted from NCBI BLAST. Alignment of the extracted sequences were performed using Clustal W and the aligned file was then used visualization with WebLogo. Download FIG S6, TIF file, 1.3 MB. Copyright © 2022 Sultana et al. 2022 Sultana et al. https://creativecommons.org/licenses/by/4.0/ This content is distributed under the terms of the Creative Commons Attribution 4.0 International license.

### Cysteine residues 54 and 95 are crucial *in vivo*.

**FIG 7:**
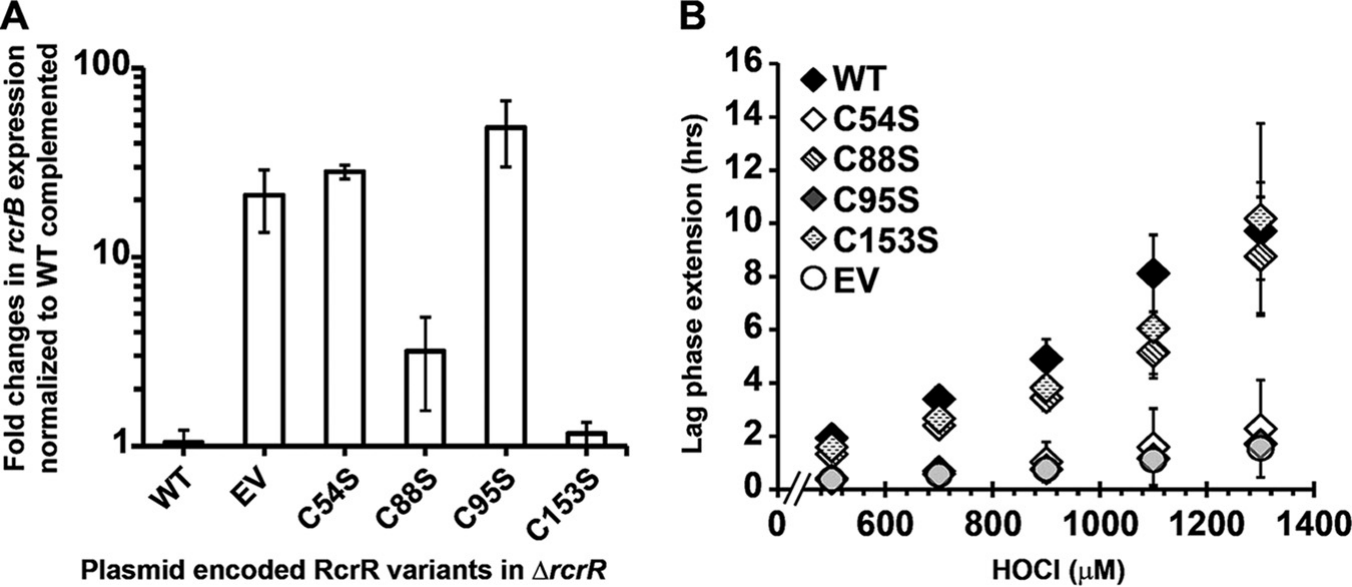
Substitution of Cys54 and Cys95 with serine results in the loss of RcrR’s repressor activity in culture. (A) Δ*rcrR* strains complemented with plasmids expressing the RcrR variants wild-type, C54S, C88S, C95S, and C153S, respectively, as well as with the empty vector (EV) pET28a were cultivated in the presence of increasing HOCl concentrations and their LPE was calculated as described in the Materials and Methods (*n* = 4, ± SD). (B) The same strains were grown to mid-log phase under nonstress conditions and *rcrB* expression was determined by qRT-PCR. Expression of RcrR-C88S and RcrR-C153S complemented the Δ*rcrR* strain whereas no complementation was observed for strains expressing RcrR-C54S and RcrR-C95S (*n* = 4, ± SD).

Our *in vitro* DNA-binding assay suggested a potential role for C54 and C95 in response to HOCl-stress. To validate their involvement in RcrR’s HOCl response *in vivo*, we performed the LPE-based growth assay using Δ*rcrR* cells expressing plasmid-encoded cysteine variants in the presence of various HOCl concentrations. Empty vector (EV) and wild-type RcrR-complemented strains served as controls. The LPE data fully support our previous findings and showed that expression of *rcrR* with serine substitutions in Cys54 or Cys95 were unable to complement the protein function, resulting in increased HOCl resistance of these strains like the EV control (Fig. 7A). In contrast, expression of the C88S and C153 variants successfully complemented the Δ*rcrR* strain.

Our transcriptomic analyses revealed RcrR represses the regulatory target genes under nonstress conditions (Fig. 3B). This phenotype could be reversed by expressing the plasmid-encoded wild-type RcrR in Δ*rcrR* cells (Fig. 7B). Similarly, expression of the C88S and C153S variants resulted in at least partial complementation of the phenotype. Complementation of Δ*rcrR* with plasmids expressing the variant proteins C54S and C95S resulted in an increased expression of *rcrB* similar to the EV control, suggesting both cysteines are also important for RcrR’s DNA-binding ability in culture. In summary, our findings indicate RcrR forms intermolecular disulfide bonds in response to RCS which involves C54 and C95. The cross-linked dimers are unable to bind to DNA and no longer repress transcription of the *rcrARB* operon.

## DISCUSSION

### RCS exposure poses a major threat to bacteria which UPEC counter by the expression of the RcrR regulon.

How pathogens regulate their behavior in response to interactions with innate immune cells is fundamental for our understanding of host colonization and the role that bacterial pathogenicity plays in infectious diseases. The production of oxidative stress by the host in response to UPEC has been reported in several independent studies along with the identification of ROS defense systems and their contribution to UPEC pathogenicity (28–30, 46, 47). UPEC face a surge in infiltrating neutrophils after arrival in the bladder (2, 4) and likely experience the largest amount of RCS in the phagosome of activated neutrophils. Particularly in inflammatory environments, infiltrating neutrophils activate the HOCl-generating enzyme MPO at concentrations in the low millimolar range, of which up to 30% leak into the extracellular surrounding (48, 49). Intriguingly, UPEC utilizes the expression of a methionine-rich peptide along with an additional methionine sulfoxide reductase system to scavenge HOCl in the periplasm (50). This unique gene cluster is highly conserved in UPEC pointing to the physiological relevance of RCS-mediated oxidative stress during UTI and the importance for UPEC to efficiently prevent RCS-mediated methionine oxidation (51). Deletion of the gene region carrying the methionine-rich peptide and the methionine sulfoxide reductase had no effect on UPEC’s growth but caused a substantial reduction in virulence (51). It is also possible that dual oxidases (Duox), which are members of the NOX family and expressed in several tissues and cell types, including mucosal barrier epithelia and uroepithelial cells of the bladder, elevate ROS/RCS level in the bladder (52). The enzymes possess a peroxidase homology domain with MPO activity vital for the host immune defense (53), and *Duox* knockdown studies revealed increased bacterial colonization and significantly higher death rates of the hosts (54–56). In fact, RclA, a member of the HOCl-specific RclR regulon, has been shown to protect E. coli from Duox-mediated oxidative stress *in vivo* (34). Thus, HOCl production and its ability to control the bacterial population in the host is physiologically relevant (57, 58), potentially providing a rationale why inflammation-associated pathogens such as UPEC have developed strategies to reduce the proteotoxic effects of HOCl.

Treatment with sublethal concentrations of HOCl resulted in a concentration-dependent growth arrest in all E. coli strains tested (Fig. 1A to C). HOCl is well known for its high microbicidal activity (16, 49, 59), which explains its role as the active ingredient in household bleach, one of the most commonly used disinfectants in medical, industrial, and domestic settings (17). The oxidant rapidly reacts with a wide range of biological molecules, including DNA, lipids, and proteins (9, 15). However, proteins are major constituents of the cell and rapidly react with oxidants like HOCl (60). Consistent with previous studies in different bacterial species (18, 25, 61–64), our RNAseq analysis of HOCl-stressed CFT073 revealed the elevated expression of various heat shock genes (Fig. 2; Table S1). The heat shock response is activated as a result of the accumulation of misfolded proteins (65), indicating proteins are also major HOCl targets in UPEC. Bacteria that lack a functional heat shock response are highly vulnerable to a variety of stresses, including HOCl exposure. It is therefore possible that oxidative protein aggregation represents one of likely several consequences of HOCl-stress that negatively affect bacterial survival (39). This is supported by the observation that the molecular chaperones Hsp33, RidA, CnoX, and polyP reduce the amount of protein aggregates in HOCl-stressed cells thereby enhancing bacterial survival (18, 39, 40, 66–68).

In comparison to EPEC and nonpathogenic E. coli strains, all UPEC isolates tested in our LPE analyses were substantially more HOCl resistant (Fig. 1A to C) implying that the UPEC pathotype is generally better equipped to deal with the negative consequences of RCS stress. We identified the *rcrARB* gene cluster as the main protection system during severe HOCl stress that enables UPEC to grow at higher HOCl concentrations (Fig. 3C and D). Our bioinformatic search revealed that the operon is predominantly found in invasive E. coli pathotypes such as APEC, AIEC and, remarkably, in ~50% of UPEC strains (Fig. 4; Fig. S3). The different pathotypes are characterized by their specific composition of horizontally acquired genetic material and the individual sets of virulence factors impact their ability to cause disease. It has been proposed that advanced resistance to phagocytosis plays an important role for the pathogenicity of APEC isolates (69). Some APEC strains have been reported to cause UTIs in humans (70), likely due to similarities in important virulence genes present in both pathotypes. It is therefore possible that APEC employ additional RCS stress defense systems, such as the RcrR regulon, to thrive in RCS-rich environments and/or survive infiltrating neutrophils. Indeed, we demonstrated that UPEC strain CFT073 is more resistant to neutrophil-mediated killing compared with the nonpathogenic E. coli K-12 strain MG1655, and that this effect is largely mediated by RcrB (Fig. 3E). Recent studies showed increased cysteine oxidation and oxidative stress in phagocytized E. coli, emphasizing the role of HOCl in microbial killing (7, 71). The same study provided strong evidence for HOCl as the main component of the oxidant mixture produced in neutrophils.

Expression of the RcrR regulon is controlled by the transcriptional regulator RcrR, which is encoded by one of the members of the operon (Fig. 3A and B). The precise biological function of the other two genes, *rcrA* and *rcrB*, is still unknown. Deletion of *rcrB* resulted in a substantial growth delay and survival defect in culture and during phagocytosis comparable with that observed in EPEC and commensal E. coli strains (Fig. 3C). This might be due to the inability of the strains to cope with the high level of proteotoxic stress requiring *de novo* synthesis of repair proteins. *rcrB* encodes a hypothetical inner membrane protein of the uncharacterized DUF417 protein family that is homologous to RclC, a member of the RclR regulon, which is also expressed in HOCl-stressed UPEC cells (Fig. 1D). The RclR regulon consists of *rclA*, *rclB*, and *rclC* and is induced through a HOCl-sensing mechanism of the transcriptional activator RclR both in lab culture and during phagocytosis, although its expression did not result in increased survival in the phagosome of neutrophils (26, 72). RclA was recently identified as a HOSCN reductase that protects E. coli from the oxidizing effects of hypothiocyanous acid, another antimicrobial oxidant generated by MPO (73). It is therefore plausible that the RcrR regulon protects UPEC from HOCl, while the RclR regulon defends against HOSCN stress. Our future studies are now directed to elucidate the role of RcrB for UPEC’s HOCl stress resistance.

### Redox regulation of the transcriptional repressor RcrR.

Bacteria have evolved numerous mechanisms on both transcriptional and posttranslational levels to fend off the toxic effects that come with the exposure to ROS/RCS. These include the conversion of ATP into the chemical chaperone polyphosphate, which was found to protect UPEC strains from HOCl-mediated protein aggregation (47), and the activation of molecular chaperones such as Hsp33, RidA, and CnoX through thiol oxidation or N-chlorination (39, 40, 66, 67, 74). Another level of protection is provided through the transcriptional activation of HOCl stress defense genes, which are directly controlled by oxidative (in-)activation of HOCl-sensing transcriptional regulators (15). One of the best characterized ROS/RCS-sensing regulators is OxyR, which is activated by cysteine oxidation and presents an important virulence factor for the pathogenicity of UPEC strain UCB34 (75). OxyR is prone to oxidation during phagocytosis (71), leading to the elevated expression of the OxyR regulon and contributing to E. coli’s ability to evolve resistance to HOCl (76). Similarly, HOCl-mediated intramolecular disulfide bond formation enables binding of the transcriptional activator RclR to the promoter to activate the transcription of the RclR regulon (26). Many of the previously identified bacterial defense systems were significantly higher expressed in our RNAseq of HOCl-stressed CFT073 cells, including Hsp33, CnoX, and members of the NemR and RclR regulons (Fig. 2).

**FIG 8:**
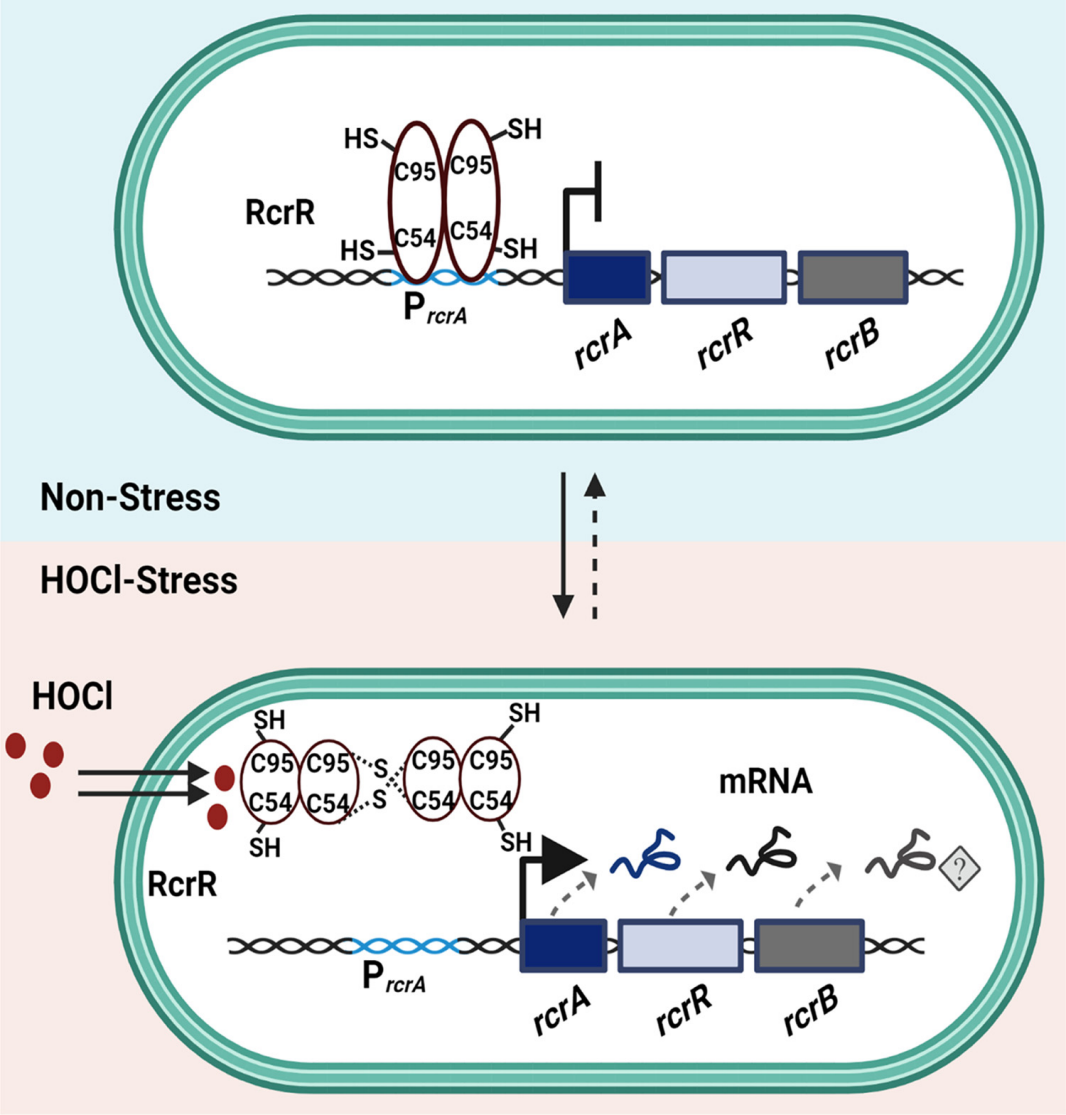
The HOCl-sensing transcriptional repressor RcrR controls the expression of the UPEC-specific genes *rcrA*, *rcrR*, and *rcrB.* Under nonstress conditions, RcrR is bound to the operator located upstream of the *rcrARB* operon and represses the expression of the three target genes. Under HOCl stress, however, as it occurs in the phagosome of neutrophils, RcrR is oxidized and forms oligomers through intersubunit disulfide bond formation leading to the inactivation of its repressor function, dissociation from the operator and derepression of the *rcrARB* transcription. Cys54 and Cys95 are important for the DNA-binding activity of the repressor. Expression of *rcrB* contributes to UPEC’s increased resistance to the antimicrobial oxidant HOCl.

We now add a novel member to the growing list of HOCl-responsive transcriptional regulators in UPEC: RcrR. RcrR belongs to the TetR family of transcriptional regulators, which are mostly alpha-helical, active as dimers (77), and share similarity in both the N-terminal DNA-binding domain and the C-terminal sensing domain with NemR, a broadly conserved HOCl-sensing repressor of the same family that is responsible for the expression of the methylglyoxal-detoxifying enzyme GloA and the N-ethylmaleimide reductase NemA (25). Although we acknowledge that additional in-depth analyses of RcrR’s inactivation mechanism are necessary to prove our hypothesis correct, we propose the following model (Fig. 8): like most redox-regulated transcriptional regulators, RcrR represses the transcription of HOCl-protective genes under nonstress conditions. Under HOCl stress, however, as it occurs in the phagosome of neutrophils, UPEC potentially senses HOCl through cysteine oxidation of RcrR. HOCl may induce the formation of disulfide-bonded RcrR oligomers between Cys54 and Cys95 causing conformational changes that inactivate the repressor and result in its dissociation from the operator and transcriptional upregulation of the *rcrARB* operon. In the presence of RCS, RcrR was shown to form intermolecular disulfide bonds both *in vitro* and *in vivo* (Fig. 6). A cysteine-free variant of RcrR did not complement the Δ*rcrR* strain and no longer responded to HOCl with the formation of disulfide-bonded oligomers (Fig. 6B), further indicating that one or more cysteines play a crucial role for the function of RcrR. Likely due to conformational changes during oligomerization, RcrR was shown to dissociate from the promoter region (Fig. 5), although the impact of RCS-mediated oxidation was rather moderate *in vitro*. In contrast, treatment of UPEC with sublethal HOCl concentrations resulted in 35- to 105-fold increased transcript levels and substantial protection from HOCl stress (Fig. 3). Likewise, deletion of *rcrR* resulted in elevated *rcrA* and *rcrB* mRNA level along with increased resistance to HOCl, suggesting that constitutive overexpression of the operon provides additional protection against HOCl (Fig. 3B and C). However, in contrast to the H_2_O_2_-sensing transcriptional regulator OxyR (78), none of the cysteines in RcrR appear to be hypersensitive. The DNA-binding activities of RcrR variants C54S and C95S were severely reduced under nonstress conditions and completely abolished in the presence of RCS (Fig. 5), providing evidence for the crucial role that both cysteines play for the at least the DNA-binding activity of RcrR. This is in contrast to NemR, which uses a completely different set of cysteines for redox-sensing and is inactivated through a thiol:sulfenamide switch (25, 79).

Given that the presence of H_2_O_2_ had no effect on the DNA binding activity, RcrR may very well be HOCl-specific much like the previously characterized transcriptional regulators RclR and HypT (24, 26, 80). This can likely be explained with HOCl’s high reactivity, as it has a 100-times shorter lifetime than H_2_O_2_, acts more local, and rapidly reacts with a variety of biomolecules (9, 23). In contrast, the thiol-specific oxidant H_2_O_2_ is orders of magnitude less bactericidal and, therefore, only kills bacteria after long exposure or at higher concentrations. Probably because they can produce this oxidant as an endogenous by-product in metabolic reactions (81), bacteria have evolved several efficient systems to eliminate H_2_O_2_ including the peroxiredoxins AhpC and AhpF, whose expression was induced in our RNAseq of HOCl-treated CFT073 (Fig. 2; Table S1). HOCl, on the other hand, is exclusively produced in eukaryotes and may therefore be perceived by bacteria as a signal for close proximity, which they utilize to adapt their responses (82).

### Is a functional HOCl response essential for UPEC colonization and disease?

Many biofilm-forming bacteria respond to changes in the environment, such as exposure to RCS, by switching from a planktonic to sessile growth (83, 84). The lifestyle change provides many survival benefits, including up to 1,000-fold increased resistance to antibiotic treatment and protection from clearance even during extensive neutrophil infiltration (85, 86). Our transcriptomic data revealed the HOCl-induced expression of various genes involved in biofilm formation (Fig. 2; Table S1), including *ydeH*, which encodes a diguanylate cyclase (87). Diguanylate cyclases are responsible for the synthesis of cyclic diguanosine monophosphate (c-di-GMP), a key regulator for biofilm formation that is involved in the regulation of cell surface-associated traits and persistent infections (88). YdeH with its N-terminal Zn^2+^-binding domain has recently been identified as the catalyst for this switch: HOCl-mediated cysteine oxidation disrupts Zn^2+^-binding of the protein and leads to increased diguanylate cyclase activity and elevated c-di-GMP level, which positively affects the production of the exopolysaccharide poly-GlcNAc and facilitates adhesion to bladder cells (82, 89). Poly-GlcNAc production is mediated by the *pgaABCD* operon, which we found to be induced in our RNAseq analysis of HOCl-stressed CFT073 (Fig. 2; Table S1), suggesting the YdeH-mediated increase in c-di-GMP indeed activates exopolysaccharide production. Many UPEC isolates express poly-GlcNAc during biofilm formation and host colonization making the polysaccharide a significant contributor to UPEC’s virulence *in vivo* (43, 90, 91). The same Zn^2+^-binding domain and equivalent HOCl-sensing mechanism has been reported in chemoreceptors such as Helicobacter pylori TlpD, which facilitates chemoattraction to HOCl sources for H. pylori and potentially explains the persistence of this pathogen in inflamed tissue (92). Exposure to HOCl also induced the expression of the curli-producing genes *csgABCEFG* (Fig. 2; Table S1), which mediate surface attachment and structural integrity in biofilm communities (93) and confer resistance to HOCl treatment (94). To establish disease, UPEC must overcome a plethora of host defenses, including neutrophilic attacks before they adhere to and invade uroepithelial cells to form intracellular biofilms (2–4). Moreover, the development of catheter associated UTIs is a result of UPEC’s ability to attach to abiotic surfaces such as catheters in an inflammatory environment. We identified the RcrR operon as a major player for UPEC’s HOCl resistance as it enables growth at otherwise toxic HOCl concentrations and contributes to resistance toward neutrophil-mediated killing (Fig. 3C to E). The gene cluster is predominantly present in E. coli pathotypes that are associated with host cell adhesion and invasion (Fig. 4; Fig. S3). A recent study reported an interdependence of resistance to ROS, biofilm formation, and pathogenicity in Proteus mirabilis, another leading uropathogen (95). Similarly, EAEC adherence to epithelial cells is stimulated by infiltrating neutrophils, presumably due to the presence of additional defense mechanisms to oxidative burst, which are therefore considered beneficial for their pathogenicity (96). However, whether UPEC has evolved the RcrR regulon as a prerequisite for their ability to overcome neutrophilic attacks, thrive in inflammatory environments, and switch from planktonic to sessile lifestyle will be subject of our future investigations.

## MATERIALS AND METHODS

### Strains, plasmids, oligonucleotides, and growth conditions.

All strains, plasmids, and oligonucleotides used in this study are listed in Table S4 in the online supplemental material. Unless otherwise mentioned, bacteria were cultivated at 37°C and 300 rpm in Luria broth (LB, Millipore Sigma) or in 3-(N-morpholino) propanesulfonic acid minimal media containing 0.2% glucose, 1.32 mM K_2_HPO_4_, and 10 μM thiamine (MOPSg) (97). Kanamycin (100 μg/mL), ampicillin (150 μg/mL), and chloramphenicol (34 μg/mL) were added when required.

10.1128/mbio.01926-22.10 TABLE S4 Strains, plasmids, and oligonucleotides used in this study. Download Table S4, XLSX file, 0.01 MB. Copyright © 2022 Sultana et al. 2022 Sultana et al. https://creativecommons.org/licenses/by/4.0/ This content is distributed under the terms of the Creative Commons Attribution 4.0 International license.

### Construction of CFT073 gene deletions.

In-frame deletion mutants were constructed using the lamda red-mediated site-specific recombination. CFT073 genes *rcrA*, *rcrR*, and *rcrB* were replaced with a chloramphenicol resistance (Cm^R^) cassette, which was resolved using pCB20 to yield the nonpolar in-frame deletion strains Δ*rcrA*, Δ*rcrR*, and Δ*rcrB*, respectively (98). All chromosomal mutations were confirmed by PCR.

### Preparation of oxidants.

The molar HOCl concentration was determined by measuring the *A*_292 nm_ of the sodium hypochlorite (Millipore-Sigma) stock solution diluted in 10 mM NaOH using ε_292_ = 350 M^−1 ^cm^−1^. The molar H_2_O_2_ concentration was quantified by measuring the *A*_240 nm_ of the stock solution diluted in 50 mM KPi buffer using ε_240_ = 43.6 M^−1^cm^−1^. N-chlorotaurine was prepared by mixing HOCl with excess of taurine (99). All oxidant dilutions were prepared fresh before each use.

### Determining HOCl susceptibility through LPE analyses.

Overnight LB cultures of the indicated strains were diluted 25-fold into MOPSg and cultivated until late exponential phase was reached (A_600 nm_ = ~1.5 to 1.8). Cultures were diluted into fresh MOPSg to an *A*_600 nm_ = 0.02 and cultivated in a Tecan Infinite 200 plate reader in the presence or absence of the indicated concentrations of HOCl and H_2_O_2_, respectively. *A*_600 nm_ measurements were recorded every 10 min for 16 h. Oxidant sensitivities of the strains tested were examined by quantifying their oxidant-mediated LPE. LPE were calculated by determining the differences in time for oxidant-treated samples to reach *A*_600 nm_ >0.3 compared with the untreated controls as described in (34).

### Survival after exposure to HOCl.

Cultures of CFT073, Δ*rcrR*, and Δ*rcrB* were either left untreated or treated with 3 mM HOCl and incubated for 30 min. Excess of HOCl was quenched by adding 5-fold molar ratio of sodium thiosulfate before samples were 10-fold diluted in PBS, spotted onto LB agar plates, and incubated overnight at 37°C.

### Gene expression analyses by qRT-PCR.

Overnight LB cultures of the indicated strains were diluted into MOPSg to an *A*_600 nm_ = 0.1 and cultivated until *A*_600 nm_ ~0.55 was reached before they were either left untreated or treated with 2.5 mM HOCl for 15 min. Then, 1 mL cells were harvested onto 1 mL of ice-cold methanol to stop transcription. After centrifugation, total RNA was prepared from the cell pellet of three biological replicates of untreated and HOCl-treated CFT073 as well as untreated Δ*rcrR*, respectively, using a commercially available RNA extraction kit (Macherey & Nagel). Remaining DNA was removed using the TURBO DNA-free kit (Thermo Scientific) and cDNA generated using the PrimeScript cDNA synthesis kit (TaKaRa). qRT-PCRs were set up according to the manufacturer’s instructions (Alkali Scientific). Transcript level of the indicated genes were normalized against transcript level of the 16S rRNA-encoding *rrsD* gene and relative fold changes in gene expression were calculated using the 2^-ΔΔC^_T_ method (100).

### RNA-seq analysis.

Samples of HOCl-treated and untreated CFT073 and Δ*rcrR* cells were collected as described before for qRT-PCR. After extraction of total RNA (Macherey & Nagel) and removal of the residual DNA using the TURBO DNA-free kit (Thermo Scientific), rRNA was depleted using the Illumina Ribo Zero Kit (Illumina) for Gram-negative bacteria. A total of 150 bp single-end sequencing was performed on an Illumina HiSeq 2500 by Novogene (Sacramento, USA). Differential gene expression analysis of three biological replicates, including normalization, was performed in the bioinformatics platform Galaxy (101). Briefly, RNAseq reads were mapped to E. coli CFT073 reference sequence (GCA_000007445.1) using HISAT2 (102). Then, the number of reads mapped to each gene were counted using featureCounts (103). Finally, differential gene expression was determined using DESeq2 (104) with an adjusted *P* value cut off *P* ≤ 0.05 and logFC cut off 1.5.

### Neutrophil-mediated killing of E. coli.

Human neutrophils were purified from fresh peripheral blood by Histopaque-1119 (Millipore Sigma) and subsequent discontinuous Percoll gradient centrifugation (105). Isolated neutrophils were resuspended in RPMI 1640 (without phenol red; Gibco) supplemented with 10 mM HEPES and 0.1% human serum albumin. The rate of bactericidal activity of neutrophils was determined and calculated by a one-step bactericidal assay as described previously (106). 1 × 10^7^ opsonized MG1655, CFT073, and Δ*rcrB* in 1 mL of RPMI supplemented with 0.1% human serum albumin were incubated in the absence and presence of 1 × 10^6^ neutrophils (final ratio of bacteria to neutrophils of 10:1). Samples were continuously rotated and incubated at 37°C for 45 min, before they were pelleted by centrifugation at 100 × *g* for 10 min. Supernatants were plated on LB agar plates to determine the number of nonphagocytized bacteria. The pellets were then washed twice with PBS, lysed in water at pH 11.0, and pelleted by centrifugation at 300 × *g* for 10 min to remove neutrophil debris. The supernatant containing the bacteria was plated on LB agar plates and incubated overnight at 37°C for CFU counts. Percent survival for each strain was calculated as the ratio of CFU in the presence of neutrophils divided by CFU in the absence of neutrophils. The IRB approval number for the neutrophil work is EA1/0104/06 (Max Planck Institute for Infection Biology, Berlin, Germany).

### Phylogenetic tree.

Genomes from 196 E. coli strains of eight pathotypes were downloaded from NCBI. A custom BLAST database was created locally with these strains. UPEC strain CFT073 genes *rcrA*, *rcrR*, and *rcrB* were identified within the custom database. A core genome alignment was constructed using Roary version 3.13.0 (107), and a maximum likelihood tree built using IqTree version 2.1.4_beta (108). The tree was visualized and annotated using Interactive Tree of Life with the pathotype and operon presence (109). Other graphs were produced using GraphPad Prism 8.4.2.

### Plasmid construction.

The *rcrR* gene was amplified from UPEC strain CFT073 genomic DNA with primers listed in Table S4 and cloned into the NdeI and BamHI sites of plasmid pET28a to generate the N-terminally His_6_-tagged RcrR expression plasmid pJUD13. RcrR variant proteins were created using the phusion site-directed mutagenesis kit (Thermo Fisher) yielding in plasmids pJUD36 (encoding RcrR-C54S), pJUD37 (encoding RcrR-C88S), pJUD38 (encoding RcrR-C95S), pJUD39 (encoding RcrR-C153S), and pJUD43 (encoding RcrR-4C-S). All constructs were verified by DNA sequencing (Eurofins).

### Expression and purification of His_6_-tagged RcrR and variants.

His_6_-tagged RcrR variant proteins were expressed in E. coli BL21(DE3). Strains were grown in 3 L LB supplemented with 100 μg/mL kanamycin until exponential phase, followed by induction with 250 μM isopropyl-β-d-thiogalactopyranoside (IPTG) for 4 h at 30°C and 200 rpm. Cells were centrifuged at 8,000 rpm for 10 min, resuspended in 50 mM NaH_2_PO_4_, 300 mM NaCl, and 10 mM imidazole (pH 8). After a 15-min incubation with 1 mg/mL lysozyme (Goldbio) and 20 μg/mL DNase (Millipore Sigma), cells were disrupted using a cell disrupter (Constant Systems LTD) and spun down for 30 min at 18,000 rpm at 4°C. The His_6_-tagged protein variants present in the supernatant were purified using Ni^2+^-NTA affinity chromatography (Goldbio) and dialyzed overnight at 4°C against 50 mM Tris-HCl (pH 7.5), 200 mM KCl, 0.1 mM MgCl_2_, 0.1 mM EDTA, 1 mM DTT and 10% glycerol (wild-type RcrR) or 50 mM potassium phosphate (pH 8), 400 mM NaCl, 2 mM DTT, 1 mM EDTA, and 5% glycerol (RcrR-C54S, -C88S, -C95S, and -C153S). Proteins were flash-frozen in liquid nitrogen and stored at −80°C.

### EMSA.

The DNA fragment (430 bp) containing the promoter region of *the rcrARB* operon (P*_rcrARB_*) was amplified by PCR using the primer pair listed in Table S4. Purified RcrR wild-type and variant proteins were exchanged into DTT-free buffer (50 mM Tris-HCl buffer [pH 7.5], 200 mM KCl, 0.1 mM MgCl_2_, 0.1 mM EDTA, and 10% glycerol) with P-30 gel chromatography columns (Bio-Rad). Increasing concentrations (0.1 to 1.25 μM) of the RcrR variant proteins were incubated with 2 ng purified P*_rcrARB_* in EMSA binding buffer (10 mM Tris-HCl [pH 7.8], 150 mM NaCl, 3 mM magnesium acetate, 10% glycerol, 100 μg/mL bovine serum albumin) for 30 min at room temperature. To study the effect of oxidants on RcrR’s DNA binding activity *in vitro*, protein variants were treated with 5-fold molar excess of NCT or H_2_O_2_ for 10 min. Excess NCT was quenched with 35 μM sodium thiosulfate prior to incubation with P*_rcrARB_* DNA fragment. DNA-binding reactions were separated by 6% TBE-polyacrylamide gel electrophoresis, stained with SYBR green (Fisher Scientific) for 30 min in the dark and fluorescence was visualized by UVP ChemStudio Plus (AnalytikJena) and quantified using ImageJ.JS.

### Nonreducing SDS-PAGE.

Purified RcrR wild-type and variant proteins were exchanged into DTT-free buffer (50 mM Tris-HCl buffer [pH 7.5], 200 mM KCl, 0.1 mM MgCl_2_, 0.1 mM EDTA, and 10% glycerol) with P-30 gel chromatography columns (Bio-Rad). A total of 10 μM proteins were oxidized for 15 min with 5- or 10-molar ratios of HOCl or H_2_O_2_ followed by addition of 1× nonreducing SDS sample buffer. To test reversibility of disulfide bond formation, HOCl-oxidized proteins were treated with 2 mM DTT. Proteins were separated by 12% SDS-PAGE and visualized after Coomassie staining.

### Western blot.

Overnight MOPSg cultures of BL21(DE3) containing plasmids expressing His_6_-tagged RcrR wild-type, His_6_-tagged RcrR-4C-S, or the empty vector control were diluted into fresh MOPSg and incubated at 37°C and 300 rpm until mid-log phase. When the cultures reached at *A*_600 nm_ ~0.35, protein expression was induced by adding 100 μM IPTG. When cells reached *A*_600 nm_ ~0.55, cultures were either left untreated or treated with 2.5 mM HOCl for 15 min. Cells equivalent to 8 mL of *A*_600 nm_ = 1 were harvested by centrifugation and incubated in 75 μL of lysis buffer (10 mM KPi [pH 6.5], 1 mM EDTA, 20% [wt/vol] sucrose, 2 mg/mL lysozyme, 50 U/mL benzonase) supplemented with 0.8 M iodoactamide for 30 min. After a quick freeze-thaw cycle and the addition of 360 μL buffer A (10 mM KPi [pH 6.5], 1 mM EDTA), cells disrupted using 0.5 mm glass beads (BioSpec Products) for 30 min at 8°C. The cell lysates were collected, proteins separated by 12% nonreducing SDS-PAGE and transferred onto a polyvinylidene difluoride (PVDF) membrane (Thermo Fisher Scientific). The membrane was blocked with Tween20-containing tris-buffered saline buffer containing 3% milk powder and 1% bovine serum albumin, incubated overnight with anti-His antibody (Cell Biolabs) and finally with horseradish peroxidase-conjugated goat anti-mouse IgG (Jackson ImmunoResearch Laboratory) for 1 h.

### Sequence analysis.

The Weblogo plot of RcrR homologs was constructed to visualize the conserved cysteine residues (110). Amino acid sequences of RcrR homologs from 31 different bacterial strains were extracted from NCBI BLAST. Alignment of the extracted sequences were performed using ClastalW and the aligned file was then used visualization with WebLogo.
