# Supplementary figures and images for: Redox-Mediated Inactivation of the Transcriptional Repressor RcrR is Responsible for Uropathogenic Escherichia coli’s Increased Resistance to Reactive Chlorine Species
Source: mBio. 2022 Sep 8;13(5):e01926-22. doi: 10.1128/mbio.01926-22 (PMC9600549; doi:10.1128/mbio.01926-22)

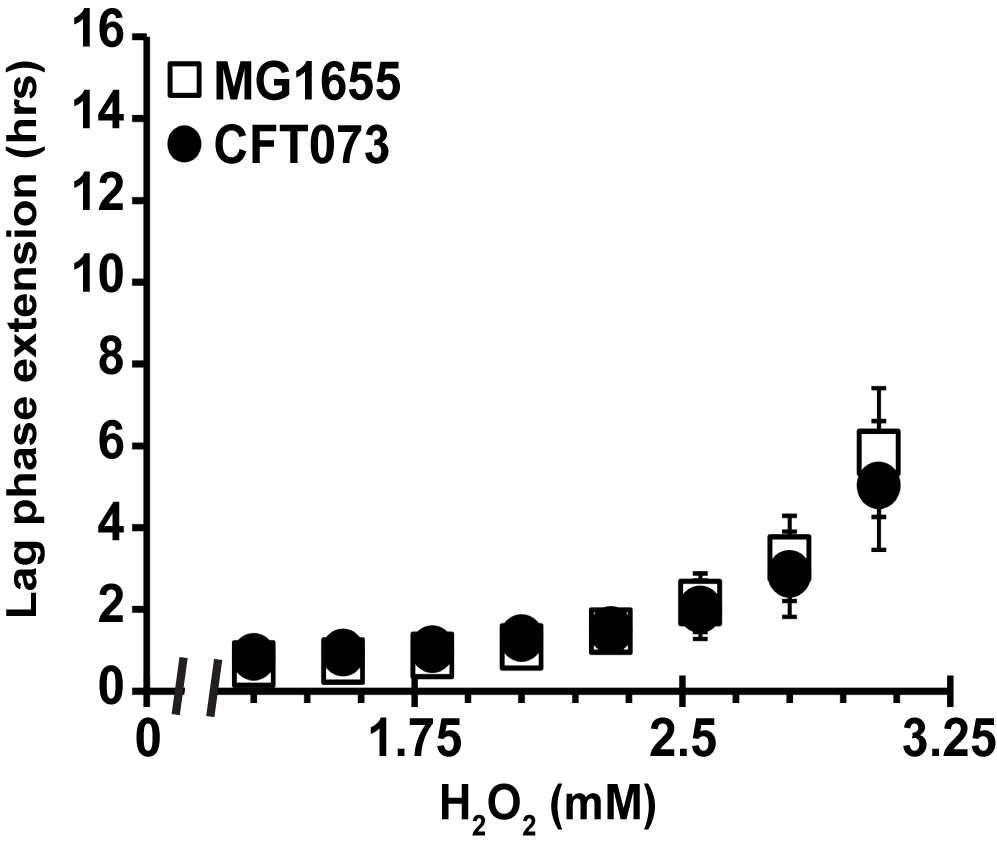

Supplement: FIG S1 [file mbio.01926-22-s0001.tif]

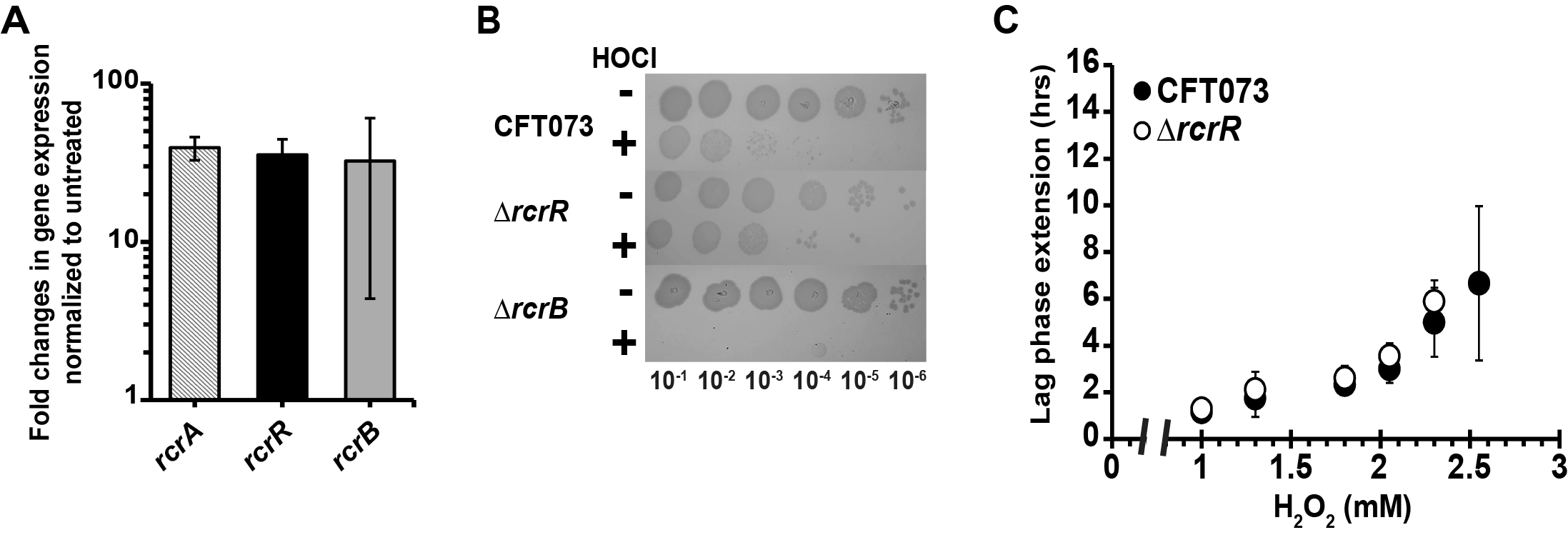

Supplement: FIG S2 [file mbio.01926-22-s0002.tif]

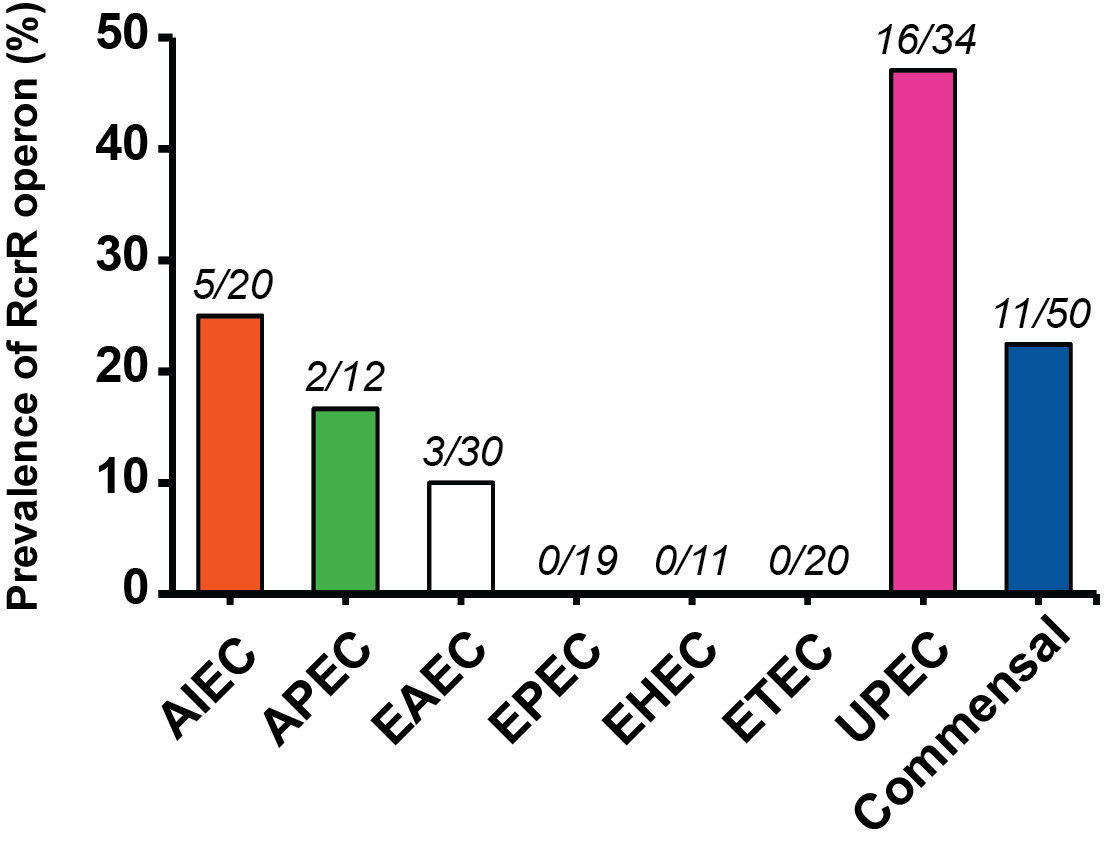

Supplement: FIG S3 [file mbio.01926-22-s0003.tif]

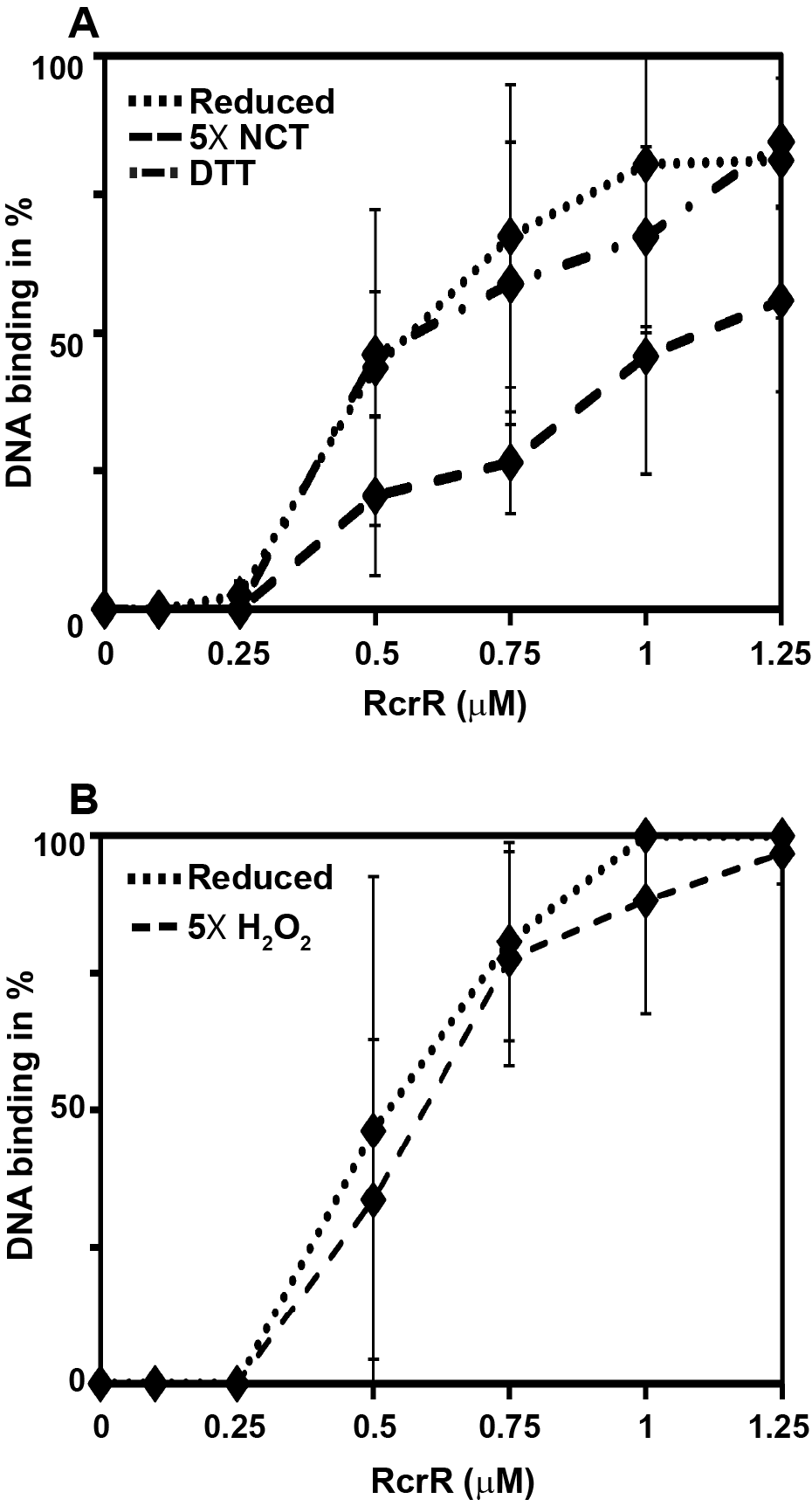

Supplement: FIG S4 [file mbio.01926-22-s0004.tif]

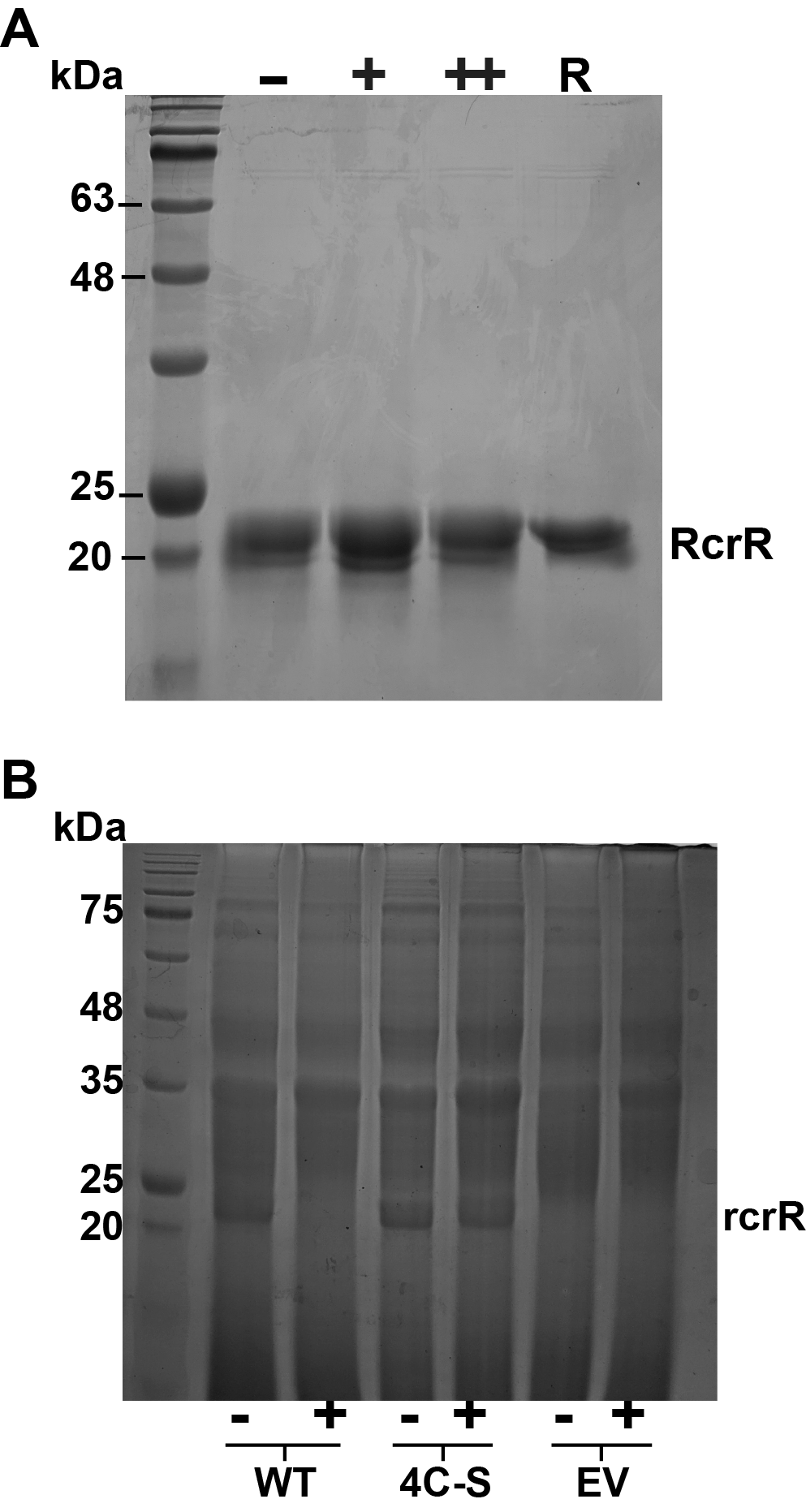

Supplement: FIG S5 [file mbio.01926-22-s0005.tif]

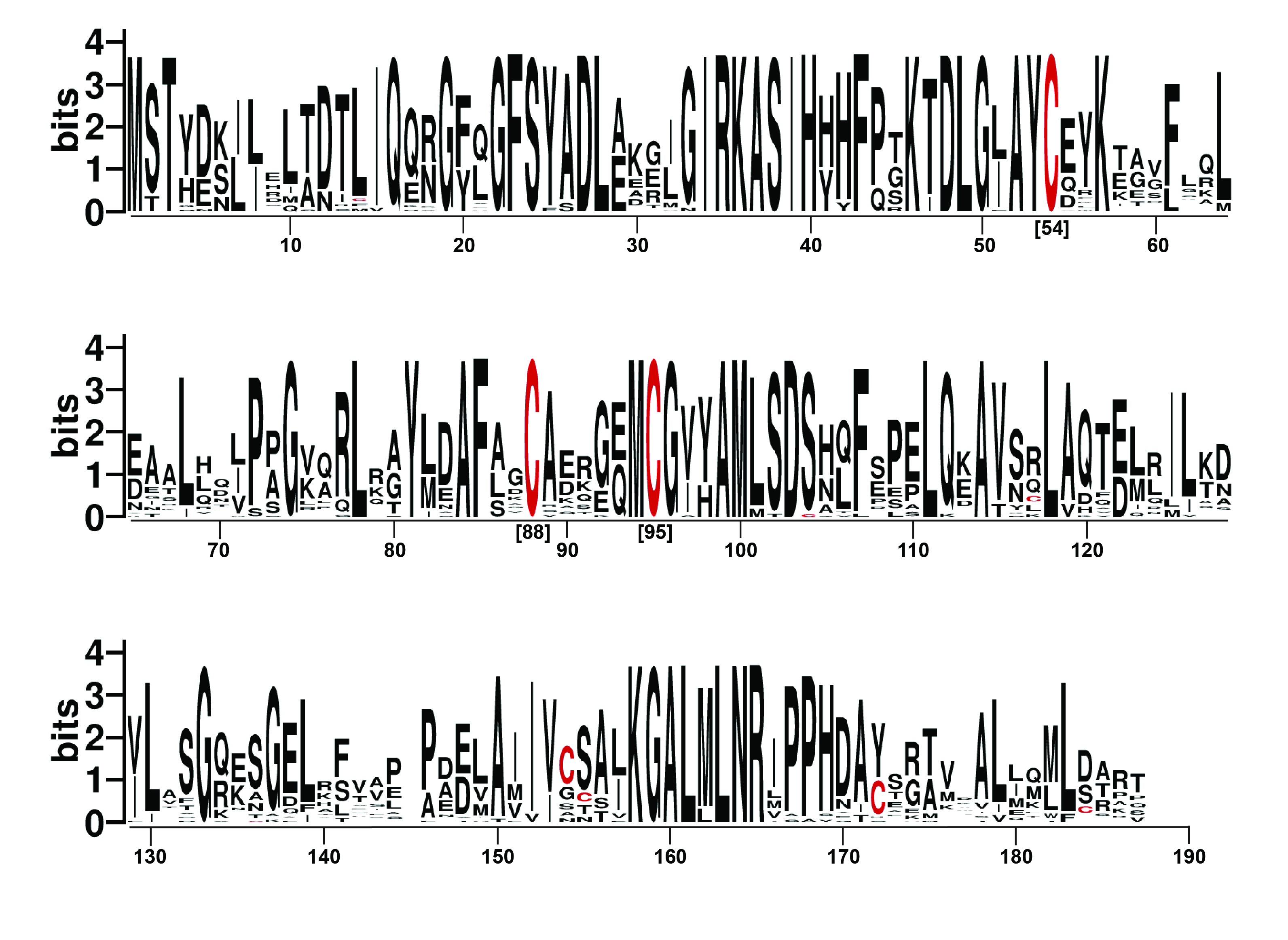

Supplement: FIG S6 [file mbio.01926-22-s0006.tif]
